# Discovering cooperation: Endogenous change in international organizations

**DOI:** 10.1007/s11558-022-09482-0

**Published:** 2022-12-14

**Authors:** Tobias Lenz, Besir Ceka, Liesbet Hooghe, Gary Marks, Alexandr Burilkov

**Affiliations:** 1 Leuphana University Lüneburg, Lüneburg, Germany; 2 Davidson College, Davidson, NC USA; 3 UNC Chapel Hill, Chapel Hill, NC USA; 4 European University Institute, Florence, Italy

**Keywords:** International organization, Regional organization, Delegation, Institutional design, Politicization, Endogenous change

## Abstract

Why do some international organizations (IO) accrete delegated authority over time while in others delegation is static or declines? We hypothesize that the dynamics of delegation are shaped by an IO’s founding contract. IOs rooted in an open-ended contract have the capacity to discover cooperation over time: as new problems arise these IOs can adopt new policies or strengthen collaboration in existing areas. This, in turn, triggers a demand for delegation. However, this logic is mediated by the political regime of the IO. In predominantly democratic IOs, delegation is constrained by politicization which intensifies as an IO’s policy portfolio broadens. These claims are tested using an updated version of the *Measure of International Authority* dataset covering 41 regional IOs between 1950 and 2019. Controlling for alternative explanations and addressing potential endogeneity across a range of model specifications, we find robust support for our argument.

**Supplementary Information:**

The online version contains supplementary material available at 10.1007/s11558-022-09482-0.

## Introduction

Why do some international organizations (IO) accrete more delegated authority over time while in others delegation is static or declines? There is remarkable variation in the delegation trajectories of IOs. Some considerably deepen delegation over time; others diminish delegation or their institutional framework barely shifts over decades of cooperation. Consider the sharply diverging delegation trajectories of Mercosur and the North American Free Trade Agreement (NAFTA), two regional organizations in the Americas that were founded in the early 1990s to liberalize trade among neighboring states. At their founding, both organizations featured intergovernmental member state bodies tasked with developing cooperative rules in the economic realm. NAFTA’s thirty-year framework was frozen in time until its recent re-negotiation as the United States-Mexico-Canada Agreement (USMCA) which tightened rules of origin but made only minimal changes to delegated institutions (Ciuriak et al., 2020). Mercosur, in contrast, has been considerably more dynamic. After the organization’s founding in 1991 with the Treaty of Asunción, its member states established a dispute settlement system with ad hoc panels in 1993, an administrative secretariat and a Joint Parliamentary Commission in 1995, and an Economic and Social Forum in 1996. In 2003, they upgraded the competences of the secretariat and in 2004 they further institutionalized the dispute settlement system by creating a Permanent Review Tribunal that can issue advisory opinions. Three years later, the members replaced the Joint Parliamentary Commission with the Mercosur Parliament and established another consultative body alongside it, the Forum of Municipalities, Federal States, Provinces, and Departments. Whereas NAFTA has witnessed just one episode of institutional reform that produced marginal shifts in its structure, Mercosur has changed its institutional framework eight times, each time boosting delegation.

What explains these contrasting delegation paths? One might ascribe these differences to the particularities of each organization. What we wish to do, instead, is to develop an explanation that can be generalized across a wide range of IOs, along the lines of Friedman’s (1953: 33) dictum “that there is a way of looking at or interpreting or organizing the evidence that will reveal superficially disconnected and diverse phenomena to be manifestations of a more fundamental and relatively simple structure.” We seek to develop a logic of institutional evolution that focuses on the internal dynamics of an IO. Specifically, we propose a theory of endogenous change in delegation that emphasizes the role of open-ended contracts in facilitating dynamic adaptation.^1^ We then test implications using an updated version of the *Measure of International Authority* dataset on delegation to independent agents in 41 regional organizations from 1950 until 2019 (Hooghe et al., 2017, 2019a). The dataset is the most comprehensive and detailed effort to date to gauge cross-sectional and temporal variation in IO delegation, and our update reveals a general upward trend through the mid-2000s followed by some slowdown over the last decade.

Scholars agree that “understanding change and development in legal rules, institutions, and procedures […] is a singularly important task” (Abbott & Snidal, 2013: 40), but it has proven easier to explain cross-sectional differences than to explain change. Many scholars share the premise that “international organizations are notoriously resistant to reform and redirection” (Barnett & Finnemore, 2004: 2). After all, institutions are generally understood as humanly devised means to make political behavior more predictable. Historical institutionalists emphasize that institutions tend to become self-reinforcing, and hence “sticky,” by structuring expectations, providing focal points for investment and generating positive feedback that locks in the status quo (Pierson, 1996, 2004). Rational choice institutionalists also stress the “striking stability and staying power of the institutional status quo,” which is reinforced because IO constitutional reform usually requires unanimity among member states following domestic ratification (Jupille et al., 2013: 5). Moreover, reforming delegation typically has uneven distributional implications, hurting some member states while favoring others (Gruber, 2000; for an overview, see Voeten, 2019: 152–54). Constructivists stress durable norms, rituals and beliefs that resist institutional change since “any efforts at change have to first overcome the power of habitual perceptions, emotions, and practices” (Hopf, 2010: 540; Barnett & Finnemore, 1999; Nelson & Weaver, 2016). Moreover, reform that empowers independent international actors cuts to the heart of national sovereignty. Any reform that diminishes state control must gain the consent of states which are normally considered “jealous guardians of political autonomy and institutional prerogatives” (Tallberg et al., 2013: 7).

Domestic politicization of international governance has further tightened constraints on delegating competences to independent IO bodies. As international governance reaches more deeply into domestic politics and restricts the ability of national governments to set policy independently, so governments will pay attention to its electoral consequences (Hooghe et al., 2019b; Walter, 2021; Zürn et al., 2012). In principle, politicization could motivate actors to increase as well as decrease IO authority, but ours are times in which politicization has chiefly mobilized national identity and the defense of national sovereignty against international governance (Börzel & Zürn, 2021; Copelovitch & Pevehouse, 2019; De Vries et al., 2021; Hooghe & Marks, 2009). While there are clear signs that nationalists push back against international organization, we know little about how this might explain either the trajectory of delegation in individual IOs or their cross-sectional variation.

Cross-sectional research has made great strides in explaining variation in IO institutional choice and design (Baccini et al., 2015; Hooghe & Marks, 2015; Jupille et al., 2013; Koremenos et al., 2001; Koremenos, 2016; Johnson & Urpeleinen, 2014; Mansfield & Milner, 2012; for an overview, see Voeten, 2019). We seek to extend this work by focusing on the temporal forces that underpin institutional evolution. Hence we contribute to a small, but growing, body of research that uses panel data to model how discrete moments of institutional reform aggregate into cumulative paths of institutional change.^2^

Our explanation shares with historical institutionalism a focus on the temporal dynamics of institutional evolution, and in this, we contribute to a burgeoning literature in international relations (Alter, 2016; Büthe, 2016; Fioretos, 2011; Hanrieder, 2015; Pierson, 1996). In line with historical institutionalism we argue that early institutional choice—in our case, the choice for an open-ended contract—conditions later decisions (Mahoney & Thelen, 2010; Streeck & Thelen, 2005). Institutional choice at an IO’s founding appears to set that IO on a distinctive path of authoritative evolution, conditioning the extent to which temporal dynamics engrain institutional stability. Whereas much of the historical institutionalist literature suggests that institutions become more deeply embedded over time, thus hampering institutional change, we show that open-ended contracts enable institutional evolution. Thus, we identify an institutional property that undermines the forces of path dependency that historical institutionalists tend to highlight.

Perhaps the most cited IR theory that focuses on the evolutionary dynamics of IO institutions is neofunctionalism. Developed and refined in the 1950s and 1960s by Haas and Schmitter, neofunctionalism emphasizes the endogenous dynamics of cooperation by positing a series of spillover mechanisms (Haas, 1958; Schmitter, 1970; Stone Sweet & Sandholtz, 1997). We build on the neofunctionalist idea of policy spillover as a spur to deeper delegation in two ways. We specify a starting condition – open-ended contracts – that enables policy spillover to be triggered, and hence makes this mechanism endogenous to an IO’s development path. And we identify a key scope condition – politicization – that constrains a functional logic of delegation. We thereby begin to address earlier criticism that neofunctionalism failed to specify “the conditions under which spillover can be expected to operate” (Keohane & Hoffmann, 1991: 20).

A theory of endogenous change is complementary with theories that emphasize exogenous change in an IO’s environment (Caporaso, 2007). Our premise is that alongside the effects of the environment, one can identify a logic of endogenous change that unfolds within an IO. Our argument is that open-ended founding contracts – those that do not stipulate a specific end goal of cooperation – enable the discovery of cooperation by allowing states to engage new policy areas or deepen cooperation in existing ones, and such policy expansion generates functional pressures for delegation. Whereas an open-ended contract enables an expansive logic of delegation, a closed-ended contract, which delineates the end goal of cooperation in precise terms, is intended to hamper policy expansion. However, even for IOs with an open-ended contract this expansive logic may not continue indefinitely. Once IO authority is perceived as domestically costly, political entrepreneurs may mobilize discontent against international governance (De Vries et al., 2021). They are likely to do so most effectively in IOs composed primarily of democratic regimes. Hence when domestic politicization hits a democratic international organization that reaches deep into domestic politics, we are likely to see a dampening of the endogenous dynamic.

We test this thesis on a sample of 41 regional IOs across the major world regions: the Americas, the Asia–Pacific, Europe, the Middle East, and Africa. Limiting the sample to authoritative regional IOs has some distinct virtues. A focus on regional, as opposed to a mix of regional and global IOs, provides a more stringent empirical test for our argument because it increases unit homogeneity, yet extends the range of alternative theories that we can consider (Sekhon, 2008: 276). With more homogenous units the unobserved biases needed to explain away a given effect tend to be larger than for a sample of widely heterogenous units (Rosenbaum, 2005). Sampling regional IOs allows us to test the effect of alternative explanations that cannot be imposed on the global level (e.g. trade interdependence) or do not vary much across global IOs (e.g. the size of IO membership, the democratic character of its member states, or asymmetry of power). Moreover, several variables of interest that exhibit only cross-temporal variation in global IOs, vary both over time and cross-sectionally in regional IOs.

The paper proceeds in three parts. The next section conceptualizes and measures IO delegation and describes the main empirical pattern: an uneven trend towards increasing delegation since 1950 which flattens in the 2010s. We then specify our expectations concerning endogenous change and its major alternatives. The final sections of the paper evaluate the validity of these expectations in fixed effects models.

## Changing delegation in international organizations, 1950–2019

Our conceptualization and operationalization of delegation follows the *Measure of International Authority* (MIA) (Hooghe et al., 2017, 2019a), and we extend the time series from 2010 to 2019. Here we summarize the chief moves that inform the measurement.

In line with convention, a regional IO is defined as an international organization composed of three or more geographically proximate states having a continuous institutional framework (Haftel, 2013; Powers & Goertz, 2011). The MIA dataset draws on the Correlates of War dataset to identify organizations that have a distinct physical location or website, a formal structure (i.e. a legislative body, executive, and administration), at least 30 permanent staff,^3^ a written constitution or convention, and a decision body that meets at least once a year. Forty-one IOs are regional in our definition, including two that no longer exist, COMECON and the first East African Community. The sample, listed in Online Appendix A,^4^ covers most states and continents, and includes all regional IOs that “have standing in international politics” (Hooghe et al., 2019a: 30).

### Conceptualization and measurement

Delegation is conventionally defined as “a conditional grant of authority from a *principal* to an *agent* that empowers the latter to act on behalf of the former” (Hawkins et al., 2006: 7, italics in original; Hooghe & Marks, 2015). States delegate authority to an IO when they empower a third party to fill in or adjudicate the details of an incomplete contract, provide expert information, select or prioritize tabled proposals, and at the authoritative high-end, propose policy initiatives, make binding decisions, or sanction contract violations (Abbott & Snidal, 1998; Bradley & Kelley, 2008; Johnson, 2013; Pollack, 2003). The principals, in this case the member states, delegate to benefit from the expertise, time and resources that agents may offer in managing policy externalities, facilitate decision-making, enhance credibility or create policy bias (Hawkins et al., 2006: 13–20). Principals retain ultimate control because they have the right to revoke delegated competences, but delegated agents enjoy a degree of autonomy, which can, and often does, change over time.

Change in delegation can have profound consequences. For one, since delegation poses a constraint on national sovereignty, delegating further competences to independent agents confronts principals with the strategic problem that these agents may shirk (see Lake, 2007), and this may compound the sovereignty dilution that international delegation always entails (Hawkins et al., 2006). Moreover, deeper delegation alters both the process and the outcomes of international cooperation: it has been shown to be associated with more productive decision making (Sommerer et al., 2022), more robust goal attainment (Gray, 2018), and a lower probability of IO death (Debre & Dijkstra, 2021).

This conceptualization of delegation is consistent with the classical IO design literature. Abbott and Snidal define delegation as “the ability to act with a degree of autonomy, and often with neutrality, in defined spheres” (Abbott & Snidal, 1998: 9). Barnett and Finnemore (2004) similarly view IOs as autonomous entities that enjoy some independence from member state control embodied, in particular, in independent international bureaucracies. Delegation features also prominently in the rational design project (Koremenos et al., 2001). And Haftel and Thompson (2006: 255) operationalize delegation—in their terms, IO independence—with a focus on bureaucracies and dispute settlement.

Delegation is related to, but distinct from, other concepts that have been used to describe the design of IOs. Especially familiar to students of regional cooperation is the distinction between intergovernmental and supranational. Whereas the former denotes IOs that are fully controlled by member states, the latter captures the capacity of “constraining the behavior of all actors, including the member states, within [specific] domains” (Stone Sweet & Sandholtz, 1997: 303; see also Helfer & Slaughter, 1997). This is akin to Boehmer et al.’s (2004: 5) concept of institutionalized IOs, which are characterized by “an ability to alter state behavior.” Both concepts are related to autonomy, defined as an “ability to operate in a manner that is insulated from the influence of other political actors—especially states” (Haftel & Thompson, 2006: 256). All these concepts share with delegation that they capture the independence of IOs from member state control. Yet, delegation is conceptually more specific because it emphasizes independence stemming from the empowerment of specific institutional actors that are given the conditional authority to perform functions on behalf of states. Therefore, delegation is often conceptualized as one of several elements of a broader characterization of IO design, such as an element of IO independence alongside autonomy and neutrality (Haftel & Thompson, 2006), or an element of legalization alongside precision and obligation (Abbott et al., 2000). Delegation, in our view, has the distinct virtue of allowing researchers to compare “completely dissimilar acts of delegation” (Brown, 2010: 144) across different domains, such as the national and the international, or different types of IOs.

The MIA operationalization that we adopt makes three moves (Hooghe et al., 2017: 107–13). First, delegation is broken down into two spheres: delegation of authoritative competences in the sphere of political decision making, and delegation in the sphere of legal adjudication. Next, each organized IO body is identified, and its composition examined for its degree of independence from member state control. And finally, each body’s role in IO decision making is evaluated.

The measure considers four types of bodies in the political sphere – IO secretariats, assemblies, executives, and consultative bodies –, and one type of body in the legal sphere – dispute settlement bodies. Organized bodies are considered independent, or non-state, when the members are primarily or wholly selected by national parliaments, regional or local governments, trade unions or business associations, or other interest groups; or when member state representation is indirect, that is, representatives are formally prohibited from receiving voting instructions from their government, or their members take an oath of independence. For each of the political bodies, its competences are assessed in setting the agenda and in making the final decision across six decision areas: the accession of new members, the suspension of members, constitutional reform, budgetary allocation, financial compliance, and up to five streams of policy making. Third-party dispute settlement breaks down into seven indicators that are conceived to jointly capture the authority of an IO’s legal dispute settlement. Four of these evaluate the extent of state control and three evaluate whether dispute settlement is supranational. All assessments are made for each year of an IO’s lifetime. All components are summed up into scales that range from 0 to 1, where 0 stands for pure intergovernmentalism and 1 for pure supranationalism, and these are then averaged to generate an annual delegation score for each IO (see Online Appendix B for more details).

The focus throughout is on formal rules that can be observed in treaties, constitutions, conventions, special statutes, protocols, and rules of procedure. The main advantage of examining formal rules is that they can be specified independently of behavior. Moreover, although states can exert influence through informal as well as formal channels, examining formal rules presents a hard case for detecting institutional change. Formal agreements impose real costs on states, are explicit and public, and are harder to change or elide because they are embedded in legal documents (Johnson, 2013). If formal international authority mattered only marginally or not at all, then one would not expect to find systematic, intelligible variation. Nor would one expect states to negotiate intensely about their content (Koremenos, 2005). Nevertheless, we do not engage institutions that exist only on paper. We investigate the formal rules and then determine whether these are translated into operating institutions to narrow the gap in coding between unrealized intention and actual practice. In other words, there needs to be evidence that institutions are indeed set up and could, at least potentially, be used.

### Empirical patterns

The most striking pattern that emerges from the data is that delegation has increased significantly over the 70 years covered in our dataset, with a rapid rise after the end of the Cold War and a slowdown over the last decade.

**Fig. 1:**
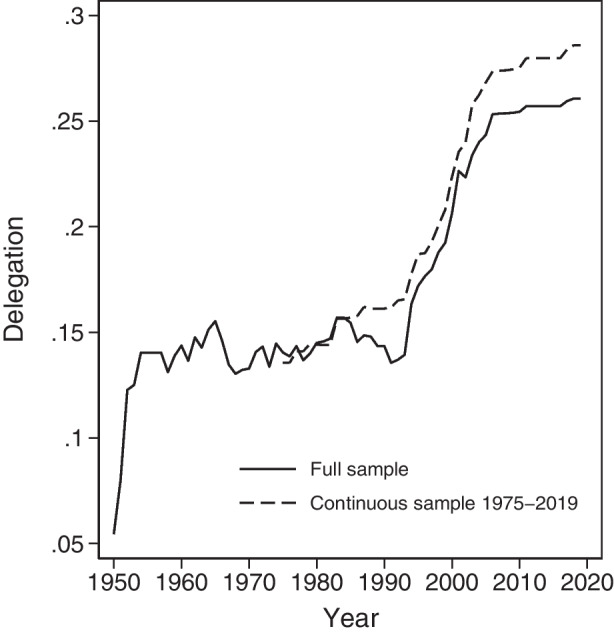
Temporal evolution of average delegation in a continuous sample from 1975 to 2019 and the full sample. *Note*: The full sample contains 41 regional IOs and the continuous sample contains 20 regional IOs from 1975 to 2019

Figure 1 shows this increase. The dashed line depicts the evolution of delegation in a continuous sample of 20 organizations from 1975 to 2019. The average delegation score more than doubled from 0.14 to 0.29. This is equivalent to adding a general secretariat with executive functions that has an exclusive right to set the policy agenda and a non-exclusive right to initiative in an additional two decision areas. The trend is less marked, but nevertheless visible, for the whole sample of IOs, because new organizations tend to start at lower levels of delegation. This upward trend envelops all but a few organizations in our dataset. However, we detect wide variation: the standard deviation is 0.15. We observe 22 year-to-year declines in delegation (of a total of 1888 year-to-year observations), and two organizations – the Economic Community of Central African States (ECCAS) and the European Centre for Nuclear Research (CERN) – experience an overall decline over the observation period.

**Fig. 2:**
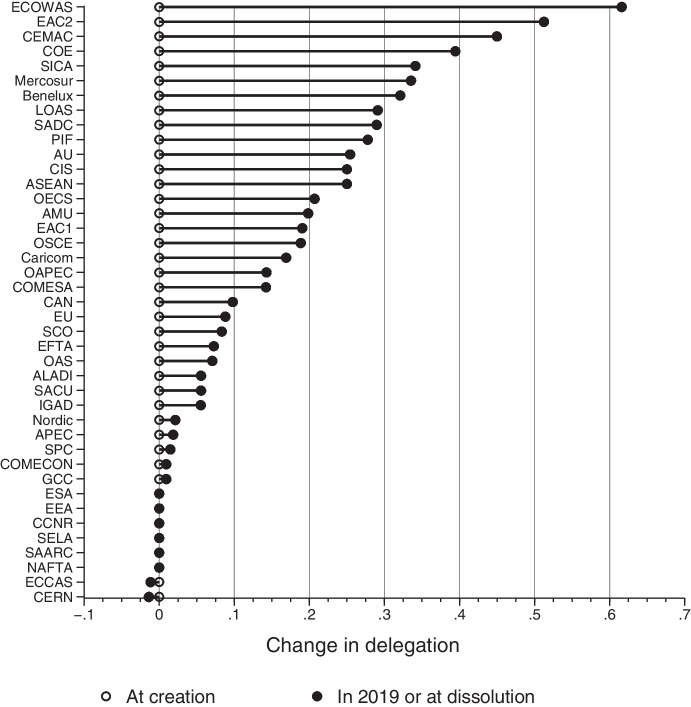
Change in delegation in 41 regional IOs, 1950–2019

Figure 2 summarizes the amount of change in delegation across the IOs in our sample during each IO’s lifetime. The single blue dots identify the six organizations that have been static according to our measure. Next up are four IOs that shift less than 0.02 on the 0–1 delegation scale. All other IOs have been more dynamic, and six IOs arrayed at the top of Fig. 2 have increased delegation by more than 0.33 on the 0–1 scale – i.e. by more than two standard deviations from the mean.

## A theory of endogenous change in IOs

What explains this variation in the delegation trajectories of IOs? Most extant theory conceives change in an IO’s institutional design as adaptation to exogenous conditions. These arguments share a view of institutions as reflecting an equilibrium among external forces that bear on an institution. Shifts in these structural conditions are hypothesized to induce changes in delegation.

This paper explores the premise that change in delegation is the result of an endogenous process that is a “product of inherent institutional properties” (Gerschewski, 2021: 3), alongside exogenous changes in its environment. Endogenous explanations specify a key mechanism whose “value is determined or influenced by an institution, and it in turn affects that institution’s development” (Rixen & Viola, 2014: 8–9). To make this stick, we need to explain how the mechanism comes about and the conditions under which it operates. Hence an argument about endogenous change in delegation should specify three elements: (1) a starting condition that sets the endogenous mechanism in motion; (2) an endogenous mechanism that determines the trajectory of delegation; and (3) a scope condition that constrains the mechanism’s operation.

**Fig. 3:**
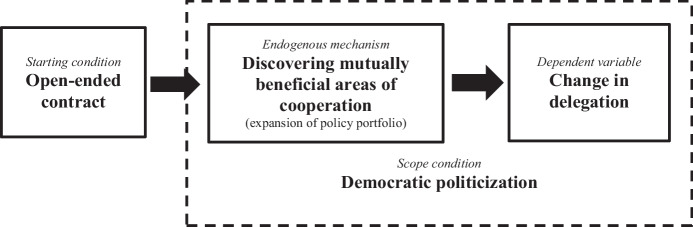
Theory of endogenous change in IOs

Open-ended founding contracts provide the starting condition that sets an IO on a distinct delegation path; policy expansion is the mechanism by which the contract produces changes in delegation; and politicization constrains the operation of the policy expansion mechanism. The argument is summarized in Fig. 3.

### Starting condition: Open-ended contracts and discovering cooperation

An IO rests on a formal contract in which states voluntarily agree to a set of formal rules for cooperation, and this becomes the starting point that sets an IO on a distinct delegation path. All such contracts seek “to structure an otherwise open future” (Wendt, 2001: 1029) and thereby mitigate uncertainty about the behavior of other participants in an exchange relationship (Koremenos, 2005). While it is impossible to specify such contracts fully, they vary in the extent to which the commitments are open-ended. At one extreme, a contract is closed in the sense that cooperation is geared towards some pre-defined and specific goal, such as establishing a free trade area in goods or monitoring a crossborder river delta. In this case, cooperation is a problem-solving exercise that helps governments tackle a well delineated transnational problem. At the other extreme, a contract involves open-ended commitments expressed in language that avoids specifying the end-destination of cooperation. In this case, cooperation entails not only problem solving but also building a common future. An IO that defines its purpose in general terms engages the transnational problems that confront a population by building and strengthening a regional community (Hooghe et al., 2019a).

Consider the two examples raised at the beginning of this paper. NAFTA approximates a closed contract. The final goal of the cooperation process is to promote a free trade area with a pre-defined scope that entails free trade in goods, services, and investment. NAFTA (now USMCA) is not intended to develop beyond this defined goal. Mercosur, in contrast, rests on an open-ended contract where the ultimate ambition is an ‘ever closer union between their peoples’ (Mercosur, 1991: preamble) – language similar to that used in the EU’s founding Treaty of Rome. This is an imprecise purpose that is infeasible, and perhaps impossible, to define in terms of a sequence of specific steps. The main protagonists in the process, such as former Argentinean President Carlos Menem (1996), have described Mercosur not only as a trade liberalization project that aims to achieve a common market and customs union but also as a “community-building process” by which Argentina and Brazil seek to overcome historic rivalries. What specifically their cooperation will look like in the future is deliberately left open.

Our understanding of contractual openness is related, yet distinct, from the notion of contractual incompleteness in contract theory (Marks et al., 2014). Contractual incompleteness is conventionally understood as the extent to which contracts “specify the full array of responsibilities and obligations of the contracting parties, as well as anticipate every future contingency that may arise throughout the course of the exchange relationship” (Cooley & Spruyt, 2009: 8). This folds two separable elements in one concept: the specificity, or precision, of commitments in *codified* policy areas and the specificity, or precision, of *future* commitments (Franck, 1988: 713–14; Hart & Moore, 1988).^5^ Contractual openness, as conceptualized here, refers solely to the latter: the extent to which the contract specifies, or details, *future* commitments. These can vary independently. It is possible for a contract to be specific on current commitments and open-ended on the future, or vice-versa. Often, organizations based on open-ended commitments start cooperation on policies for which commitments can be relatively detailed. For example, the contract underpinning the Organization of Eastern Caribbean States (OECS) contains open-ended commitments regarding the ultimate purpose of the organization alongside policy commitments regarding the creation of a common market detailed in nearly thirty pages of dense text. Importantly, the creation of a common market is only seen as an initial step in a longer process towards creating a “closer union among the peoples of the East Caribbean” (OECS, 1968: preamble).

We hypothesize that open-ended contracting has a marked effect on the course of delegation in an IO. Unlike closed-ended contracts, open-ended contracts endow participants with flexibility because they entail vague objectives that can be reached in various ways and are therefore easier to adjust to unforeseen circumstances (Hart & Moore, 2008). In an era of interdependence, the opportunities for mutually beneficial cooperation between states are vast but they do not reveal themselves automatically to governments. As Thompson aptly observes, open-ended contracts structure the environment in such a way that it does not “disclose the alternatives available or the consequences of those alternatives” (Thompson, 2003: 9). Therefore, international cooperation under open-ended contracts involves continuous bargaining in the discovery of mutually beneficial cooperation. Open-ended contracts are “loose contracts,” in Hart and Moore’s (2004: 3) terminology, in which there is much to bargain over after the contract has been signed. At stake is not merely the issue of compliance with initial commitments, but rather the translation of a broad and open-ended ambition into concrete policies. What policies should governments subject to common rules as they seek to realize an “ever closer union”? Open-ended contracts induce dynamics that Chayes and Chayes have described as a “creative enterprise through which the parties not only weigh the benefits and burdens of commitment but explore, redefine, and sometimes discover their interests” (Chayes & Chayes, 1993: 180). This discovery process is facilitated because these contracts generate the flexibility for an IO, over time, to learn how to adapt its policies to changing needs: they may engage new policy areas as the transnational problems that member states confront evolve; or they may deepen cooperation in existing policy areas when this appears beneficial to member states. Closed-ended contracts, in contrast, constrain that flexibility because the purpose of cooperation is clearly specified; member states are less likely to discover cooperation and may indeed wish to limit it.

The connectedness among policy fields generates pressures for policy expansion in any IO (Haas, 1958; Schmitter, 1969), but only IOs resting on open-ended contracts can potentially widen their policy portfolio without renegotiating the IO’s mandate, whereas IOs with closed-ended contracts are hemmed in by their mandate and have to find other ways to deal with policy pressures. Hence, we expect different rates of policy expansion in the two types of IOs:

- *H*_*1*_* (starting condition): Open-ended contracts enable an expansive logic of policy change over time, while closed-ended contracts inhibit it.*

### Causal mechanism: Policy expansion and delegation

How do open-ended contracts induce distinct delegation trajectories? We posit a causal mechanism of change by which the expansion of an IO’s policy portfolio – spurred by open-ended contracting – generates a demand for delegation to handle the increasing complexity of decision making. Since a change in delegation usually requires treaty reform, member state representatives will be the actors who ultimately decide, but pressures for delegation change can be brought to bear by a variety of actors—including international bureaucrats, nonstate actors, alongside national bureaucrats or politicians—as they experience a mismatch between the institutional status quo and the perceived need for cooperation. As Pierson (2000: 483) aptly puts it, “More prevalent and complex political activity places growing demands on decision makers […] and [enhances] the need to delegate decisions.” There is abundant evidence for this functional logic in the expansion of civil services, courts, and agencies within national states. Summarizing research on delegation within the state, Moe (2012: 17) observes that “In complex policy areas, the value of agency […] will tend to be higher, and the optimal level of independence higher.”

We argue that the empowerment of third-party agents occurs along four primary functions that IOs perform. The first is setting the policy agenda. Delegation to an independent agent may help structure iterative and recurrent decision making. A delegated agent can help avoid issue cycling by framing the agenda (Hawkins et al., 2006: 16–17; Pollack, 2003: 84–85). The scope for issue cycling is likely to be larger in open decision-making contexts where the issue space is multidimensional. As Tsebelis (2002: 154) notes, the increasing dimensionality of decision making adds to the number of voters who have the deciding vote in an otherwise tied outcome. Delegating agenda setting power to a non-state actor is one solution, and in the field of international organization, this usually involves an independent secretariat with the authority to draft legislative proposals (Hawkins et al., 2006; Pollack, 2003).

The second function is providing information. As an IO’s policy portfolio broadens, so does the need for unbiased information. Arrow (1974: 53–56) points out that while an organization can acquire vastly more information than can any individual, this information must be carefully structured to be of use in decision making. Non-state agents may be valuable in retrieving, filtering, and disseminating information that would be expensive for a state to produce (Bradley & Kelley, 2008; Koremenos, 2008; Pollack, 2003). Non-governmental organizations (NGOs), for example, may have a comparative advantage in providing local knowledge and in publicly monitoring member state commitments (Tallberg et al., 2014: 754–55). Moreover, a reputation for detachment from any one country—cultivated by an independent IO secretariat—may be useful in gaining the trust of national interlocutors and in retrieving unbiased information (Beyers & Trondal, 2004; Egeberg, 1999; Hooghe, 2005). For each of these reasons, independent non-state actors may have informational access and expertise that becomes more valuable as an IO’s policy portfolio grows. Providing unbiased information and expertise is therefore one of the primary “gains from specialization” (Hawkins et al., 2006: 13).

Third, policy expansion intensifies the problem of monitoring and enforcement. The more complex the policy environment, the greater the scope for contending interpretations of whether a particular behavior is a rule violation. Jurisdiction to interpret the meaning of the law is a basic court function. “Since the principals themselves disagree on what the contract implies, they cannot instruct the agent on exactly how to decide on the issue(s) under dispute. Principals, therefore, go to considerable lengths to select (or create) impartial agents with relatively high autonomy” (Hawkins et al., 2006: 18; Kono, 2007; Koremenos, 2008: 168–69). Hence it makes sense to empower an independent panel or standing court to arbitrate disputes and enforce rulings by fine, sanction, or retaliation (Alter, 2008; Carrubba & Gabel, 2017; Dworkin, 1988).

The fourth function is legitimation. IOs used to rest almost exclusively on the legitimacy of national governments to take decisions in the international realm. However, when an IO’s policy portfolio expands and diplomacy becomes international policy with tangible domestic repercussions, legitimacy cannot derive only from the quality of an IO’s output, but needs to be complemented by procedural and input legitimacy (see Scharpf, 1999). Delegating participatory/consultative or even decision-making competences to non-state actors composed of parliamentarians or other stakeholders is one way to do this (Rocabert et al., 2019). Moreover, states may hope to enhance IO legitimacy by creating parliamentary assemblies and other non-state bodies that domestic audiences regard as appropriate forms of institutionalization (Lenz et al., 2019).

These dynamics suggest that as an IO comes to have a broader policy footprint its member states will be induced to empower non-state actors:

- *H*_*2*_* (mechanism): The expansion of an IO’s policy portfolio drives increases in delegation.*

### Scope condition: Democratic politicization and the dampening of an expansive logic

The responsiveness of democracies to public opinion can constrain delegation when an IO becomes popularly contested. If politicization is perceived to diminish a democratic leader’s electability, they may respond by dampening, or even reversing, delegation (De Wilde et al., 2017; Hooghe & Marks, 2009; Hutter et al., 2016; Voeten, 2022). In democratic settings, dissatisfaction with government policies is more likely to be aired in the press (Stier, 2015). Experimental research shows that when IOs are contested, negative framing has a greater impact on public opinion than positive framing (Dellmuth & Tallberg, 2021).

Research on the European Union, the most supranational IO in the world, has shown that politicization can be a game changer in the dynamics of international cooperation in a democratic setting (Hooghe & Marks, 2009). In the face of overwhelming functional pressures in the wake of the financial crisis or the migration crisis, it has proven very hard indeed for the European Union to respond by deepening delegation (Börzel & Risse, 2018; Hooghe & Marks, 2019; Schimmelfennig, 2018; Scipioni, 2018). This is not an exclusively European phenomenon. In many countries, IOs have been politicized by nationalist populist parties and politicians who defend national sovereignty against international cooperation (Copelovitch & Pevehouse, 2019; Hooghe et al., 2019b; Walter, 2021). Mercosur, the Andean Community, CARICOM, and SICA experienced bouts of politicization following the third wave of democratization (Ribeiro Hoffmann, 2015; Riggirozzi, 2015). As Hurrelmann and Schneider (2015: 255) note, “large scale politicization in the late 1980s and early 1990s had the effect of discouraging political elites from pursuing further integration initiatives, and this in turn made politicization recede.” This has been particularly consequential with respect to international courts. Voeten (2021: 152, 162) counts 28 episodes of backlash against eleven different international judicial bodies since 1990, of which the large majority have been initiated by leaders in democratic countries.

We hypothesize that politicization is especially constraining in IOs that operate on a broad policy front and where delegation under open-ended contracting is advanced (Tallberg & Zürn, 2019; Zürn, 2018). As IOs become more independent from member state control and expand their influence, they are likely to get push-back, particularly in a competitive democratic setting. When politicization hits, electoral politics come strongly into play (Broz et al., 2021; De Vries et al., 2021; Flaherty & Rogowski, 2021; Goldstein & Gulotty, 2021).

- *H*_*3*_* (scope condition): The expansive logic of delegation is dampened by politicization in democratically dominated IOs.*

## Determinants of change in international organizations: A multivariate analysis

We test our expectations on endogenous change in multivariate analysis. We begin by describing relevant variables and model choices.

### Operationalization

Our theory posits four key variables: the nature of an IO’s contract, policy scope, politicization, and democratic IO.^6^

*Contract* is operationalized by assessing whether an IO’s foundational contract formulates its objectives, i.e. future commitments, in specific or open-ended terms.^7^ We code a contract as closed-ended (value = 0) if its purpose is to achieve a fixed purpose of interstate cooperation under clearly specified conditions. For example, the objectives of the European Space Agency (ESA) are precisely formulated as follows: “for exclusively peaceful purposes, cooperation among European states in space research and technology and their space applications” (ESA, 1975: article 2). A contract is open-ended (value = 1) if its purpose is to achieve broad-ranging cooperation that is only vaguely specified, for example, as a “community of peoples,” “political federation,” or in terms of “unity” or a “common identity.” The best-known example is the European Union with its diffuse and open-ended commitment to an “ever closer union among the peoples of Europe” (EU, 1957: preamble). The Caribbean Community’s 1973 Treaty is another example of an open-ended contract calling for economic cooperation as a step to “fulfil the hopes and aspirations of their peoples for full employment and improved standards of work and living” (CARICOM, 1973: article 4). While the treaty focuses on economic cooperation, it commits member states to “take all appropriate measures” for “the achievement of a greater measure of economic independence and effectiveness of its Member States” (Articles 4 & 5). In the absence of an existing measure, we developed the coding scheme ourselves and tested its reliability with independent coders who produced convergent scores. Table 1 below shows the distribution of IOs by type of contract in their first and last year in the time series. While an organization can change its contract over time, this is rare. Three IOs—Benelux, CARICOM, and IGAD—shift from closed- to open-ended contracts. Results hold if we use the original or revised contracts.

**Table 1:** IOs by contract

	First year	Last year
Closed-ended	16	13
Open-ended	25	28

N = 41 IOs over 1950–2019 (or last year in dataset)

*Policy Scope* is estimated as the number of policies in which an IO is engaged in a given year across a list of twenty-five policies (Hooghe et al., 2017, 2019a). Our expectation is that a change in *Policy Scope* leaves a material footprint in the budget, a legal document (a protocol, an annex, a convention), or in the creation, expansion, or elimination of an institution (e.g. a commission, a working group, a directorate, a crisis management mechanism, or a high-profile position). We use eight legal, financial, and organizational indicators to assess whether there is tangible evidence that an IO’s portfolio encompasses a particular policy.

*Politicization* estimates the salience and divisiveness of debate over an IO. Media coverage of protests directed at an IO is an accessible and plausible indicator for contestation about an IO (Beyeler & Kriesi, 2005; Tarrow, 2005). We implement an algorithm developed by Tallberg et al. (2014) for annual media coverage of protests/demonstrations directed at an IO in the LexisNexis database. *Politicization* is calculated as a three-year moving average of the number of mentions (log10) that combine the word *protestor* or *demonstrator* with the IO name.^8^

We construct a binary measure of *democratic IO* by using the *Varieties of Democracy* (V-dem) estimates for each member state of an IO (Coppedge et al., n.d.) to calculate the average democratic quality of an IO. We follow the recommendation of V-Dem in using 0.5 as the cut-off point (Lührmann et al., 2018), hence an IO is coded as democratic if the average V-Dem democracy score for its members in any given year is higher than 0.5. In robustness analyses, we also use the continuous V-Dem measure of democracy.

#### Contending explanations

Our argument differs from arguments that link IO change to factors that can be plausibly conceived as exogenous. Here we evaluate three contenders: power asymmetry, foreign policy heterogeneity, and trade interdependence.

Some argue that large power asymmetry may put downward pressure on IO delegation because hegemonic actors reject constraints on their national sovereignty (Gruber, 2000; Lipscy, 2017; Stone, 2011). As Abbott and Snidal (2000: 448) note, “forms of legalization that involve limited delegation […] provide the crucial basis for cooperation between the weak and the strong.” Others contend that large power asymmetries may facilitate building stronger independent institutions, because hegemonic states are more willing to provide public goods as they need not worry about an unfavorable distribution of institutional benefits (Kindleberger, 1973; Martin, 1992; Mattli, 1999). *Power asymmetry* is the ratio of the material capabilities of the most powerful member state to the sum of all other members. The measure combines total population, total Gross Domestic Product (GDP) and military expenditure.^9^

Preference divergence is likely to make states less inclined to cede authority to non-state bodies because their institutional ideal points are likely to be further apart and the fear that independent institutions are used to work against a member state’s preferences is larger (Keohane, 1984). We operationalize this liberal line of argumentation in two ways. First, we evaluate how foreign policy congruence/incongruence among the member states of an IO affects their willingness to delegate (Voeten, 2021). *Preference Heterogeneity* uses data gathered by Bailey et al. (2017) who estimate country-year ideal points that reflect the extent to which two states vote differently in the UN Assembly for the period 1946–2019.

Finally, we evaluate how congruence on economic interests among an IO’s member states affects their willingness to delegate. *Trade interdependence* is a proxy for congruent economic interests since trading partners could be expected to have a convergent interest in deepening trade rules (Haftel, 2013; Martin, 1992; Mattli, 1999). Our preferred measure is *Intra‐regional trade*, which captures a region’s total trade as a proportion of member countries’ total trade, though we also report two alternative operationalizations.

#### Controls

We control for the size of an IO’s membership on the premise that “centralization of information is […] increasingly valuable with larger numbers” (Koremenos et al., 2001: 789). *Members* is the natural log of the number of member states in a given IO-year. Second, we control for the political regime of an IO’s members on the expectation that an IO with democratic member states will be less fearful of exploitation (Mansfield et al., 2002, 2008; Simmons, 2009; Tallberg et al., 2016). *Democracy* draws on *Varieties of Democracy* estimates (Coppedge et al., n.d.) to calculate the annual average democracy score for an IO. Third, we control for *GDP-per-capita* on the premise that more affluent states transact more across borders and may have greater demand for international cooperation. A fourth control is *GDP dispersion*, the standard deviation in GDP per capita among members, on the expectation that the more economically heterogeneous the member states of an IO, the greater the benefit of empowering the IO to mediate conflicts (Carnegie, 2014; Koremenos et al., 2001: 785–86). Finally, *Cold War* is a dummy with value of zero from 1991 on the ground that the dissolution of two-power hegemony created new demands for regional cooperation.

Tables including descriptive statistics and bivariate correlations among the variables can be found in Online Appendix D.

### Estimation strategy

A theory of endogenous institutional change entails a two-step estimation strategy whereby an institutional property triggers a causal mechanism (step 1) that affects institutional development (step 2). In our test, we first investigate whether an open-ended contract leads to policy expansion in a regional organization over time (H_1_). We then test whether policy expansion drives change in delegation (H_2_) and whether politicization acts as a brake on delegation in democratic IOs once policy scope expands (H_3_).

We test our argument using time-series cross-sectional data. The analysis faces a number of inferential threats (e.g. longitudinal and group-wise heteroskedasticity and correlation of standard errors) which complicate the selection of appropriate estimators (Beck & Katz, 2011). Since our main goal is to examine change over time, we use fixed effects models. This allows us to estimate change in the levels of policy scope and delegation within IOs over time while accounting for IO-specific factors that are time invariant. All of our models use one-year lagged independent variables and, to control for factors changing every year or specific to a certain period in an IO’s existence, we include either a year count or the age of the IOs and a Cold War dummy. Finally, to address autocorrelation we also conduct analyses with a lagged dependent variable.

A theory of endogenous change posits a causal sequence in which variables build on each other, but a statistical test requires that at least one variable is treated as exogenous. We theorize the IO’s founding contract as the starting condition of an endogenous process. We measure *Contract* at an IO’s founding moment, with considerable temporal distance (between 1 and 82 years) to the institutional change that we seek to account for. Change in the contractual nature of IOs is rare (three out of 41 regional IOs display it) and we run all our analyses also without these organizations. Yet, sceptics may contend that even contract, though temporally distant from the outcome of interest, may be endogenous to the evolutionary institutional dynamics we observe because policy makers may *intend* an IO to evolve over time and, *therefor*e, design an open-ended contract. We address this concern about potential endogeneity between contract and policy scope through an instrumental variable approach, using a measure of historical ties – the extent to which an IO’s founding members have a shared past of colonialism or prior statehood – that is temporally even more distant to our outcome variable and clearly exogenous to the decision to establish an IO, or to write an open- or closed-ended contract. As we show, *Historical ties* is a strong instrument that meets the exclusion restriction.

### Results

We start our analysis by taking a descriptive look at the trajectories in delegation of different types of IOs. Our core claim is that IOs with an open-ended founding contract are more likely to display an expansive path of delegation than are those that rest on a closed-ended contract, and that this logic operates via the differential growth of an IO’s policy portfolio. 

**Fig. 4:**
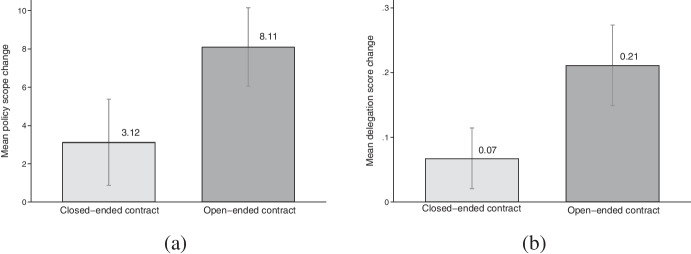
Mean change in policy scope (**a**) and delegation (**b**) based on type of contract, 2019 (or last year in the dataset) - year of founding

Figure 4 shows how these two types of IOs differ with regard to the average change in policy scope (4a) and in delegation (4b), measured as the difference in the respective values between the last and the first year in the dataset. The divergence between open-ended and closed-ended contracts is striking. IOs that were set up with a closed contract gained, on average, 3.1 policies on a 25-point scale over the course of their lifetime, while those with an open-ended contract increased their policy scope, on average, by 8.1 policies. The pattern is similar for delegation: an average increase of 0.07 for IOs with closed-ended contracts against 0.21 for IOs with an open-ended contract on a 0–1 scale. These differences are substantively important and highly statistically significant under controls in a cross-sectional analysis.^10^ These patterns suggest that IOs with open-ended contracts display a much higher dynamic in delegation than IOs with a closed-ended contract, lending initial support to our core claim.

**Table 2:** Explaining change in policy scope (time-series cross-sectional analysis with fixed effects)

	(1)	(2)	(3)	(4)	(5)	(6)	(7)
VARIABLES	Baseline	Extended baseline	Main model	With trade intensity	With trade introversion	With CINC	Excluding Benelux, Caricom, IGAD
Contract _t-1_	1.646	1.448	1.237	1.427	1.405	1.560	
	(1.097)	(1.092)	(1.149)	(1.208)	(1.173)	(1.185)	
Age _t-1_	0.039***	0.043***	0.002	0.006	0.007	-0.008	-0.018
	(0.010)	(0.010)	(0.047)	(0.047)	(0.045)	(0.049)	(0.050)
Contract _t-1_ * Age _t-1_	0.140***	0.145***	0.136***	0.133***	0.133***	0.133***	0.152***
	(0.033)	(0.034)	(0.032)	(0.033)	(0.033)	(0.036)	(0.031)
Members _t-1_			1.944	1.637	1.621	1.819	2.276
			(2.187)	(2.297)	(2.312)	(2.237)	(2.477)
Democracy _t-1_			7.288	6.831	6.798	7.271	7.401
			(6.417)	(6.224)	(6.087)	(6.923)	(6.768)
GDP per capita _t-1_			-0.459	-0.497	-0.509	-0.278	-0.029
			(0.958)	(0.955)	(0.944)	(0.994)	(0.965)
GDP dispersion _t-1_			0.329	0.392	0.387	0.214	0.170
			(0.492)	(0.503)	(0.542)	(0.516)	(0.542)
Power asymmetry _t-1_		-0.223	-0.452**	-0.437**	-0.440**	-0.221	-0.483*
		(0.158)	(0.221)	(0.208)	(0.208)	(0.226)	(0.243)
Trade interdependence _t-1_		-0.026	-0.054	-0.001	-0.124	-0.052	-0.082
		(0.083)	(0.073)	(0.003)	(2.223)	(0.074)	(0.069)
Cold War _t-1_			-0.839*	-0.821*	-0.835	-0.860	-1.169**
			(0.495)	(0.485)	(0.527)	(0.534)	(0.505)
Preference heterogeneity _t-1_		-0.770	-1.023	-0.769	-0.802	-0.503	-1.161
		(1.016)	(1.130)	(1.081)	(1.041)	(1.051)	(1.190)
Constant	4.781***	6.605***	2.387	1.624	1.761	1.676	3.937
	(0.451)	(2.425)	(5.385)	(5.110)	(5.346)	(5.386)	(6.535)
Observations	1887	1715	1708	1708	1708	1591	1564
R-squared	0.541	0.522	0.558	0.554	0.554	0.527	0.566
Number of IOs	41	41	41	41	41	41	38

Robust standard errors in parentheses; *GDP per capita* and *GDP dispersion* are standardized for easier interpretation.

*** *p* < 0.01, ** *p* < 0.05, * *p* < 0.1

A more rigorous test needs to employ the full temporal variation that our data affords. We start by investigating whether open-ended contracting leads to policy expansion in regional organizations over time (H_1_). Table 2 presents the results of fixed effects models with policy scope as the dependent variable using various specifications. We theorize that an open-ended contract creates the flexibility for an IO, *over time*, to learn how to adapt its policies to changing needs. Hence, this opens the door for policy spillover. In contrast, a closed contract constrains that flexibility. The key point is that time matters differently for IOs with different types of contracts. Thus, it makes sense to model contract dynamically so we can observe separately the effect of time and the theorized accelerator effect of an open-ended contract. We do this by interacting the type of contract with the age of an IO.^11^

**Fig. 5:**
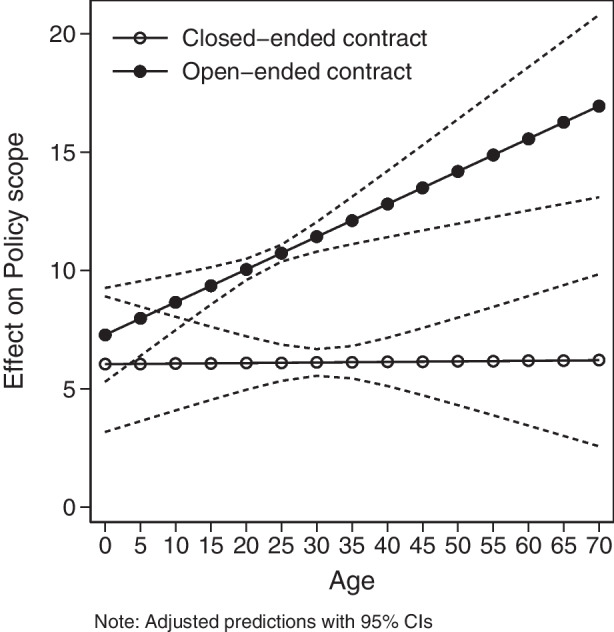
Effect of *Contract* on *Policy scope* at various age of the IO

The first two columns of Table 2 show a baseline model with only the key variables of interest (model 1) and an extended baseline model with fewer controls (model 2). Model 3 is our main model. Regardless of which controls we use, the main results are remarkably consistent – the interaction term has the correct positive sign and is statistically significant, suggesting that open-ended contracts enable IOs to discover cooperation over time while closed-ended contracts do not. To better visualize the interaction effect, Fig. 5 shows predictive margins for open- and closed-ended contracts for various levels of IO age.^12^ The results strongly support H_1_. None of the controls, except for *Power asymmetry* and *Cold War,* reach conventional levels of statistical significance. As expected, IOs characterized by higher power asymmetries among their members have experienced less policy increase. The end of the Cold War seems to have facilitated policy expansion, although this finding is not consistently significant across model specifications.

The main results appear robust across various specifications. Specifically, we employ alternative measures of trade such as trade intensity and trade introversion (models 4 and 5), the standard CINC measure for power asymmetry (model 6), and we exclude from the analysis the three IOs that experienced a contract change (model 7). In addition, we ran the main analysis using random effects and with a lagged dependent variable (see Online Appendix, Table F1).^13^ The substantive interpretation of our findings is the same.^14^ In short, there is considerable evidence that open-ended contracts lead to policy expansion over time.

What if policy makers design contracts at founding *with the intention to* enable or restrict policy scope expansion over time? We address the potential endogeneity of contract by instrumenting contract with a variable that is clearly exogenous to both the choice of IO contract and policy scope. *Historical ties* captures whether the founding members of an IO have a shared political past of colonialism or prior statehood (e.g. as a federation). *Historical ties* reflect bonds that were forged prior to the design of the IO, so there is no doubt that it is exogenous to the decision to create an IO, or to write an open- or closed-ended contract. Moreover, as we detail in Online Appendix E, *Historical ties* is a strong instrument in that member states that have historical ties are more likely to sign open-ended contracts (relevance of the instrument). At the same time, we leverage the three IOs that witness a change in their founding contracts to suggest that historical ties affect policy expansion only through contract and not through other types of interactions, such as trade or migration that could influence policy scope directly, therefore fulfilling the assumption of the exclusion restriction.

A two-stage fixed effects model, presented in Online Appendix E (Table E1), tests our conjecture. In the first stage, *Historical ties* is a statistically strong predictor of *Contract*. According to the Stock-Yogo test, we can reject the null hypothesis of weak instrumentation at the strictest threshold of 10 percent. In the second stage, *instrumented Contract* is significantly associated with change in policy scope. Two-stage estimation is almost always less efficient than ordinary least squares estimation (Bartels, 1991), but here there is no loss of statistical power.

**Fig. 6:**
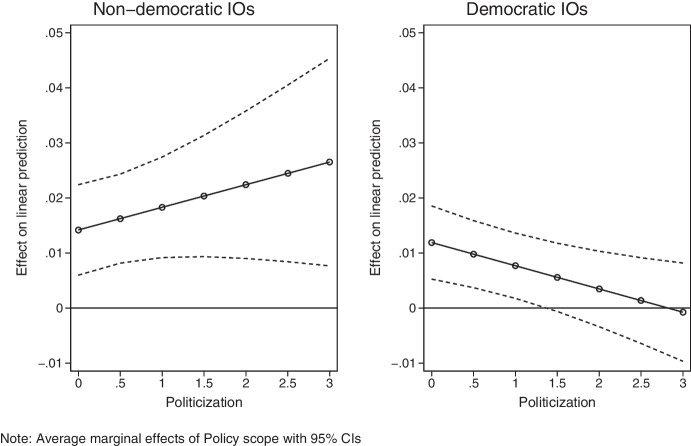
Effect of *Policy Scope* on *Delegation* at various levels of politicization for non-democratic IOs and democratic IOs

In the second step of our analysis, we test whether the expansion of an IO’s policy portfolio drives increases in delegation (H_2_) and whether politicization puts a brake on this process among democratically dominated IOs (H_3_). Since we expect politicization to put downward pressure on further delegation to the extent that an IO’s policy portfolio expands, we use a three-way interaction between politicization, policy scope, and a dummy variable for whether an IO is predominantly democratic.^15^ Table 3 presents fixed-effects regression results with *Delegation* as the dependent variable. As with policy scope in Table 2, we also report results using trade intensity and trade introversion (models 4 and 5), the CINC measure for power asymmetry (model 6), and we exclude from the analysis the three IOs that experienced a contract change (model 7). Figure 6 plots the average marginal effects of a one unit change in policy scope over various levels of politicization, and we plot the interaction separately for non-democratic and democratic IOs.^16^

**Table 3:** Explaining change in delegation (time-series cross-sectional analysis with fixed effects)

	(1)	(2)	(3)	(4)	(5)	(6)	(7)
VARIABLES	Baseline	Extended baseline	Main model	With trade intensity	With trade intro-version	With CINC power asymmetry	Excluding Benelux, Caricom, IGAD
Policy scope _t-1_	0.018***	0.018***	0.014***	0.014***	0.014***	0.015***	0.015***
	(0.003)	(0.004)	(0.004)	(0.004)	(0.004)	(0.004)	(0.004)
Politicization _t-1_		0.005	-0.025	-0.025	-0.028	-0.025	0.003
		(0.058)	(0.041)	(0.041)	(0.041)	(0.037)	(0.042)
Policy scope _t-1_ * Politicization _t-1_		0.003	0.004	0.004	0.004	0.005	0.003
		(0.004)	(0.003)	(0.003)	(0.003)	(0.003)	(0.003)
Democratic IO _t-1_		0.001	-0.030	-0.030	-0.032	-0.014	-0.009
		(0.045)	(0.044)	(0.044)	(0.043)	(0.045)	(0.044)
Democratic IO _t-1_ * Policy scope _t-1_		-0.003	-0.002	-0.002	-0.002	-0.004	-0.004
		(0.005)	(0.004)	(0.004)	(0.004)	(0.004)	(0.004)
Democratic IO _t-1_ * Politicization _t-1_		0.132**	0.098**	0.098**	0.103**	0.089**	0.079**
		(0.064)	(0.041)	(0.042)	(0.042)	(0.036)	(0.039)
Democratic IO _t-1_ * Policy scope _t-1_ * Politicization _t-1_		-0.010**	-0.008**	-0.008**	-0.009**	-0.008**	-0.007**
		(0.004)	(0.003)	(0.003)	(0.003)	(0.003)	(0.003)
Members _t-1_			-0.034	-0.034	-0.037	-0.052**	-0.028
			(0.030)	(0.031)	(0.032)	(0.025)	(0.037)
Power asymmetry _t-1_			0.006	0.006	0.006	0.008**	0.005
			(0.004)	(0.004)	(0.004)	(0.004)	(0.004)
GDP per capita _t-1_			-0.038**	-0.038**	-0.040**	-0.033*	-0.046**
			(0.017)	(0.017)	(0.017)	(0.017)	(0.018)
GDP dispersion _t-1_			0.010	0.010	0.007	0.007	0.013
			(0.009)	(0.010)	(0.010)	(0.009)	(0.009)
Year _t-1_			0.003***	0.003***	0.003***	0.003***	0.002***
			(0.001)	(0.001)	(0.001)	(0.001)	(0.001)
Trade interdependence _t-1_			0.000	0.000	-0.041	0.000	0.000
			(0.001)	(0.000)	(0.059)	(0.001)	(0.001)
Cold War _t-1_			0.010	0.010	0.008	0.014	0.009
			(0.009)	(0.009)	(0.009)	(0.009)	(0.010)
Preference heterogeneity _t-1_			-0.041**	-0.041*	-0.045**	-0.041**	-0.041**
			(0.019)	(0.022)	(0.021)	(0.019)	(0.019)
Constant	0.027	0.023	-4.912***	-4.912***	-5.234***	-4.992***	-4.604***
	(0.028)	(0.038)	(1.587)	(1.587)	(1.730)	(1.561)	(1.610)
Observations	1,887	1,887	1,708	1,708	1,708	1,591	1,564
R-squared	0.455	0.535	0.572	0.572	0.574	0.562	0.576
Number of IOs	41	41	41	41	41	41	38

Robust standard errors in parentheses; *GDP per capita* and *GDP dispersion* are standardized for easier interpretation.

*** *p* < 0.01, ** *p* < 0.05, * *p* < 0.1

Table 3 and Fig. 6 support H_2_ and H_3_. Specifically, increased policy scope leads to more delegation (H_2_) as can be seen by the positive and highly statistically significant coefficient for *Policy scope* in Table 3*.* However, and in line with H_3_, politicization dampens delegation in an IO with democratic member states. A three-way interaction finds that at higher levels of politicization (exceeding 1.5) the effect of policy scope on delegation loses significance (Fig. 6). For example, a one standard deviation increase in policy scope (5.8 on a 1-to-25 scale) in a democratic IO without politicization is associated with almost a half standard deviation increase in delegation (+ 0.07 on a 0-to-1 scale), whereas the same increase in an IO with 30 protest events a year (1.5 on the X-axis) is associated with an effect on delegation that is indistinguishable from zero. In line with our prior, politicization has no dampening effect on delegation in an IO with non-democratic member states.^17^

Among the controls, *Preference Heterogeneity* and *GDP per capita* and the standard CINC measure for power asymmetry are statistically significant at the 0.05 level. As expected, divergent foreign policy preferences among IO members exert downward pressure on IO delegation. Change in *GDP per-capita* is negatively related and *Power asymmetry (CINC)* positively related to change in delegation, though the substantive effect for both is negligible. Perhaps the greatest surprise is that the association between trade interdependence and delegation is insignificant across the board. This fits poorly with functionalist theories that expect pressure from trade links on deepening delegation (Keohane, 1984; Stone Sweet & Brunell, 1998; for a more extensive treatment, see Hooghe et al., 2019a).

What about endogeneity here? Could it be that instead of policy expansion driving changes in delegation, the relationship is the reverse? After all, neofunctionalists already recognized potential recursivity between policy spillover and the role of supranational agents (Haas, 1958), and recent studies associate delegation with the expansion of an IO’s policy portfolio (see, for example, Haftel & Hofmann, 2017). To test for a reverse relationship between *Delegation* and *Policy scope*, we conduct a procedure proposed by Dumitrescu and Hurlin (2012) to detect Granger causality in panel datasets. This allows us to evaluate whether past values of *Delegation* forecast (i.e. Granger-cause) present values of *Policy scope* and indicates whether this is true for a subset of IOs. For the 30 ROs^18^ in which we observe sufficient change in both *Delegation* and *Policy scope* during the observation period, the test reveals that for the overwhelming majority *Delegation* does not Granger cause *Policy scope*. However, the test suggests a recursive relationship between policy expansion and delegation for 4 IOs.^19^ We then re-ran our main analysis with *Delegation* as the dependent variable but excluded the 4 IOs identified above. The main results are virtually identical to those reported in Table 3 (see Online Appendix, Table F2).

In all, the analysis suggests that the institutional evolution of delegation in regional IOs has limited exogenous roots, but that there is a powerful endogenous dynamic that begins at foundation with an open-ended contract which provides the IO and its members greater capacity to discover cooperation over time, and which in turn spurs delegation. However, this functional engine starts to sputter in democratically dominated IOs as an IO becomes politicized in domestic contestation.

To further probe our results, we ran our analysis using random effects and with a lagged dependent variable (see Online Appendix, Table F2).^20^ The results of these additional analyses are broadly consistent.^21^

## Conclusion

This paper develops a theory of endogenous change to explain variation in the delegation trajectories of regional IOs. Our argument emphasizes the legacy of contractual choice at an organization’s founding moment which sets the stage for a distinct developmental path for an IO’s policy portfolio and, subsequently, for delegation. Because it does not specify the end-result, an open-ended contract facilitates flexibility in discovering cooperation. By contrast, a closed-ended contract limits such flexibility. This, we believe, has major consequences for delegation. An expanding policy portfolio then provides the causal mechanism that translates an open-ended founding contract into deeper delegation. We suspect that what drives this is a desire to reap efficiency gains or overcome legitimation constraints that arise when international cooperation becomes more complex.

However, we have reason to believe that the expansive logic of delegation does not continue indefinitely. If IO cooperation among democratic states becomes embroiled in domestic contestation, the functional logic of delegation is weakened. The constraining effect of politicization has been documented in the European Union, and we suggest that this can be generalized to other democratic IOs.

The proposed explanation draws on neofunctionalism and historical institutionalism. The causal mechanism from policy expansion to deepening delegation is analogous to the neofunctionalist notion of functional spillover developed by Ernst Haas. We refine neofunctionalism in two ways. First, we propose that reforms to deepen delegation are not only motivated by a desire to reap efficiency gains but also by a wish to put extensive international cooperation on a sounder footing of legitimacy. And second, we identify the key starting condition that can trigger policy spillover, the IO contract, as well as a key scope condition, politicization.

Our theory shares with historical institutionalism a focus on the temporal dynamics of institutional evolution. It stresses the cross-temporal incentives and constraints that policy makers face as a result of institutional choices at an IO’s outset. In line with historical institutionalism we argue that early institutional choices—in this case, the choice for an open-ended contract—condition later ones. This suggests it is useful to qualify the “punctuated equilibrium model” (Krasner, 1984), which depicts institutional evolution as sharp bursts of exogenously induced change followed by longer periods of stasis. And it refines historical institutionalism’s close association of institutional stability with path dependency by revealing how distinctive forms of path dependency may coexist with contrasting degrees of stability and dynamism.

Our findings raise several questions for future research. One such puzzle concerns the sources of support for IO authority. We know a lot about those who oppose globalization and international governance (Broz et al., 2021; De Vries et al., 2021; Dellmuth et al., 2022; Hooghe & Marks, 2018), but we know less about the social bases of pro-IO constituencies (Bornschier et al., 2021; Hooghe & Marks, 2022) and the conditions under which the latter counter-mobilize (De Wilde et al., 2019). An empirical challenge here is to develop measures that reliably capture the full repertoire of mobilization and counter-mobilization.

The negative association between politicization and IO authority in democratic countries is contextual, but how this will evolve in the future is unclear. Contemporary anti-IO politicization reflects a populist backlash from social groups that have suffered a decline in life chances as a result of globalization (Kriesi et al., 2008; Owen & Johnston, 2017; Goldstein & Gulotty, 2021; Walter, 2021). It is possible that this backlash is more time-bound than we can now foresee. According to some observers, one effect of COVID-19 might be that labor shortage and deglobalization reduce inequality in western democracies, which may perhaps lessen hostility against IOs (Flaherty & Rogowski, 2021).

Our purpose here is to shed light on the endogenous dynamics of IO development. We see our concern with IOs as political institutions as complementary to research that conceives IOs in networks of diverse actors, including states, corporations, social movements, and other IOs. Indeed, network theorists stress that one must pay attention to the characteristics of the units in explaining the relative strength of their ties and why some actors are more central than others (Lazer, 2011: 64). One perspective complements the other.

## Supplementary Information

Below is the link to the electronic supplementary material.

Supplementary file1 (PDF 774 KB)

Supplementary file2 (ZIP 200 KB)

## Data Availability

The dataset used in the current study will be made available in a Dataset repository. Data that is already publicly available is referenced accordingly throughout the article and in the Online Appendix.

## Footnotes

1 This argument builds on Marks et al. (2014).

2 Comparativists have also noted that “the vast literature that has accumulated provides us with precious little guidance in making sense of processes of institutional change” (Mahoney & Thelen, 2010: 2).

3 *Yearbook of International Organizations*, multiple years.

4 The Online Appendix is available on the *Review of International Organization*s' webpage.

5 The influential legalization concept, for example, focuses primarily on the precision of commitments in existing policy areas, which “narrows the scope for reasonable interpretation” (Abbott et al., 2000: 412).

6 Online Appendix C provides more detail on the operationalization of all variables.

7 The theoretical literature sometimes treats contractual incompleteness as a continuous phenomenon but in the absence of prior measures, we opt for a binary operationalization. A recent example of measuring the precision of treaty substance in continuous terms is Gastinger and Schmidtke (2022).

8 The measure is quite strongly correlated with an estimate of the salience of an IO derived from a count of references to the IO in Google (r = 0.75).

9 This operationalization amends and updates the CINC series in the COW dataset (Singer, 1988), which ends in 2012. Robustness checks that use the original CINC measure (up to 2012) produce consistent results.

10 In the cross-sectional analysis (not shown here) we use average scores for the covariates in Table 2 for each IO (a) in 1950, or the year of their establishment, until 2019 (b).

11 Time is modelled here as year-creation + 1 (whereby creation is the date of creation or 1950, whichever is later). Even IOs that survived WWII had to be rebooted to resume cooperation.

12 This figure is constructed using the results from model 3.

13 The random effects model gives the average of the between and within results, so it also captures the cross-sectional variation in our data. Hausman tests show that model fit is significantly better with fixed effects than random effects.

14 In additional cross-sectional tests, not shown here, we confirm that the exogenous variables cannot account for the nature of an IO’s founding contract, which allows us to exclude the possibility that they exert causal effects through contract.

15 The results of three-way interactions are simpler to interpret if at least one of the variables is dichotomous. However, as a robustness check we run our analysis with a continuous V-Dem measure of democracy (Online Appendix F, Table F2 and Figure F1).

16 This figure is constructed using the results from model 3.

17 This is consistent with studies that suggest an autocratic government may employ an IO, such as the Shanghai Cooperation Organization or the Arab League, to shore up their rule (Ambrosio, 2008; Debre, 2021).

18 Eleven IOs with little or no change in either of these variables are dropped from the analysis: CCNR, CERN, CIS, COMECON, EAC1, EEA, ESA, NAFTA, SAARC, SACU, and SELA.

19 These are PIF, SPC, GCC, and ECCAS.

20 A Hausman test shows that the fixed effects model provides a better fit to the data.

21 The three-way interaction of politicization, democratic IO, and policy scope loses significance in the most demanding model with a lagged dependent variable.

## References

[CR1] Abbott K, Snidal D (1998). Why states act through formal international organizations. Journal of Conflict Resolution.

[CR2] Abbott K, Snidal D (2000). Hard and soft law in international governance. International Organization.

[CR3] Abbott, K. & Snidal, D. (2013). Law, legalization, and politics. In Dunoff, J. & Pollack, M. (eds.), *Interdisciplinary perspectives on international law and international relations: The state of the art* (pp. 33–56). Cambridge: Cambridge University Press. 10.1017/CBO9781139107310.003

[CR4] Abbott K, Keohane R, Moravcsik A, Slaughter AM, Snidal D (2000). The concept of legalization. International Organization.

[CR5] Alter, K. (2008). Delegation to international courts: Self-binding vs. other-binding delegation. *Law and Contemporary Problems, 71*(37), 37–76. https://www.jstor.org/stable/27592221

[CR6] Alter, K. (2016). The evolution of the international judiciary. In Fioretos, O., Falleti, T. G. & Sheingate, A. (eds.), *Oxford handbook of historical institutionalism* (pp. 590–610). Oxford University Press. 10.1093/oxfordhb/9780199662814.013.35

[CR7] Ambrosio T (2008). Catching the "Shanghai Spirit": How the Shanghai Cooperation Organization promotes authoritarian norms in central Asia. Europe-Asia Studies.

[CR8] Arrow K (1974). The limits of organization.

[CR9] Baccini L, Dür A, Elsig M (2015). The politics of trade agreement design: Revisiting the depth-flexibility nexus. International Studies Quarterly.

[CR10] Bailey MA, Strezhnev A, Voeten E (2017). Estimating dynamic state preferences from United Nations voting data. Journal of Conflict Resolution.

[CR11] Barnett M, Finnemore M (1999). The politics, power, and pathologies of international organizations. International Organization.

[CR12] Barnett, M., & Finnemore, M. (2004). *Rules for the world: International organizations in global politics*. Cornell University Press.

[CR13] Bartels L (1991). Instrumental and ‘quasi-instrumental’ variables. American Journal of Political Science.

[CR14] Beck N, Katz JN (2011). Modelling dynamics in time-series-cross-section political economy data. Annual Review of Political Science.

[CR15] Beyeler M, Kriesi H (2005). Transnational protest and the public sphere. Mobilization.

[CR16] Beyers, J., & Trondal, J. (2004). Nation-states “hit” Europe: Ambiguity and representation in the European Union. *West European Politics,**27*(5), 919–942. 10.1080/0140238042000283265

[CR17] Boehmer C, Gartzke E, Nordstrom T (2004). Do intergovernmental organizations promote peace?. World Politics.

[CR18] Bornschier S, Häusermann S, Zollinger D, Colombo C (2021). How ‘us’ and ‘them’ relates to voting behavior: Social structure, social identities, and electoral choice. Comparative Political Studies.

[CR19] Börzel T, Risse T (2018). From the Euro to the Schengen crises: European integration theories, politicization, and identity politics. Journal of European Public Policy.

[CR20] Börzel T, Zürn M (2021). Contestations of the liberal international order: From liberal multilateralism to postnational liberalism. International Organization.

[CR21] Bradley, C. & Kelley, J. (2008). The concept of international delegation. *Law and Contemporary Problems, 71*(1), 1–36. https://www.jstor.org/stable/27592220

[CR22] Brown RL (2010). Measuring delegation. Review of International Organizations.

[CR23] Broz JL, Frieden J, Weymouth S (2021). Populism in place: The economic geography of the globalization backlash. International Organization.

[CR24] Büthe, T. (2016). Historical institutionalism and institutional development in the EU: Development of supranational authority over government subsidies (state aid). In Rixen, T. & Viola, L. A. (eds.), *Historical institutionalism and international relations* (pp. 37–67). Oxford University Press. 10.1093/acprof:oso/9780198779629.003.0002

[CR25] Caporaso J (2007). The promises and pitfalls of an endogenous theory of institutional change: A comment. West European Politics.

[CR26] CARICOM. (1973). *Treaty of Chaguaramas establishing the Caribbean Community including the CARICOM Single Market and Economy*. Chaguaramas, July 4.

[CR27] Carnegie A (2014). States held hostage: Political hold-up problems and the effects of international institutions. American Political Science Review.

[CR28] Carrubba CJ, Gabel M (2017). International courts: A theoretical assessment. Annual Review of Political Science.

[CR29] Chayes A, Chayes AH (1993). On compliance. International Organization.

[CR30] Ciuriak, D., Dadkhah, A., & Xiao, J. (2020). Quantifying CUSMA: The economic consequences of the new North American trade regime. C.D. Howe Institute Working Paper, February 21.

[CR31] Cooley, A., & Spruyt, H. (2009). *Contracting states: Sovereign transfers in international relations*. Princeton University Press.

[CR32] Copelovitch M, Pevehouse J (2019). International organizations in a new era of populist nationalism. Review of International Organizations.

[CR33] Coppedge, M., et al. (n.d.) V-Dem Dataset v10. *Varieties of Democracy (V-Dem) Project*.

[CR34] De Vries CE, Hobolt SB, Walter S (2021). Politicizing international cooperation: The mass public, political entrepreneurs, and political opportunity structures. International Organization.

[CR35] De Wilde P, Leupold A, Schmidtke H (2017). Introduction: The differentiated politicisation of European governance. West European Politics.

[CR36] De Wilde, P., Koopmans, R., Merkel, W., Strijbis, O., & Zürn, M. (2019). *The struggle over borders: Cosmopolitanism and communitarianism*. Cambridge University Press.

[CR37] Debre M (2021). Legitimation, regime survival, and shifting alliances in the Arab League: Explaining sanction politics during the Arab Spring. International Political Science Review.

[CR38] Debre, M., & Dijkstra, H. (2021). Institutional design for a post-liberal order: Why some international organizations live longer than others. *European Journal of International Relations,**27*(1), 311–339. 10.1177/1354066120962183

[CR39] Dellmuth, L., & Tallberg, J. (2021). Elite communication and the popular legitimacy of international organizations. *British Journal of Political Science,**51*(3), 1292–1313. 10.1017/S0007123419000620

[CR40] Dellmuth L, Scholte JA, Tallberg J, Verhaegen S (2022). The elite-citizen gap in international organization legitimacy. American Political Science Review.

[CR41] Dumitrescu EI, Hurlin C (2012). Testing for Granger non-causality in heterogeneous panels. Economic Modelling.

[CR42] Dworkin, R. (1988). *Law’s empire*. Belknap Press.

[CR43] Egeberg M (1999). Transcending intergovernmentalism? Identity and role perceptions of national officials in EU decision-making. Journal of European Public Policy.

[CR44] ESA. (1975). *Convention for the establishment of a European Space Agency*. Paris, May 30.

[CR45] EU. (1957). *Treaty establishing the European Economic Community*. Rome, March 25.

[CR46] Fioretos O (2011). Historical institutionalism in international relations. International Organization.

[CR47] Flaherty TM, Rogowski R (2021). Rising inequality as a threat to the liberal international order. International Organization.

[CR48] Franck T (1988). Legitimacy in the international system. American Journal of International Law.

[CR49] Friedman, M. (1953). The methodology of positive economics. In M. Friedman (Ed.), *Essays in positive economics* (pp. 3–43). Chicago University Press.

[CR50] Gastinger M, Schmidtke H (2022). Measuring precision precisely: A dictionary-based measure of imprecision. Review of International Organizations, Online First..

[CR51] Gerschewski J (2021). Explanations of institutional change: Reflecting on the "missing diagonal". American Political Science Review.

[CR52] Goldstein J, Gulotty R (2021). America and the trade regime: What went wrong?. International Organization.

[CR53] Gray J (2018). Life, death, or zombie? The vitality of international organizations. International Studies Quarterly.

[CR54] Gruber, L. (2000). *Ruling the world: Power politics and the rise of supranational institutions*. Princeton University Press.

[CR55] Haas, E. B. (1958). *The uniting of Europe: Political, social, and economical forces, 1950–1957*. Stanford University Press.

[CR56] Haftel Y (2013). Commerce and institutions: Trade, scope, and the design of regional economic organizations. Review of International Organizations.

[CR57] Haftel Y, Hofmann S (2017). Institutional authority and security cooperation within regional economic organizations. Journal of Peace Research.

[CR58] Haftel Y, Thompson A (2006). The independence of international organizations: Concept and appplications. Journal of Conflict Resolution.

[CR59] Hanrieder T (2015). The path-dependent design of international organizations: Federalism in the World Health Organization. European Journal of International Relations.

[CR60] Hart O, Moore J (1988). Incomplete contracts and renegotiation. Econometrica.

[CR61] Hart, O., & Moore, J. (2004). *Agreeing now to agree later: Contracts that rule out but not rule in*. Research Discussion Paper No. 2032, Harvard Institute of Economics. 10.2307/1912698

[CR62] Hart O, Moore J (2008). Contracts as reference points. Quarterly Journal of Economics.

[CR63] Hawkins, D., Lake, D., Nielson, D., & Tierney, M. (2006). Delegation under anarchy: States, international organizations, and principal-agent theory. In Hawkins, D., Lake, D., Nielson, D. & Tierney, M. (eds.), *Delegation and agency in international organizations* (pp. 3–38). Cambridge University Press. 10.1017/CBO9780511491368.002

[CR64] Helfer L, Slaughter AM (1997). Toward a theory of effective supranational adjudication. The Yale Law Journal.

[CR65] Hooghe L (2005). Several roads lead to international norms, but few via international socialization: A case study of the European Commission. International Organization.

[CR66] Hooghe L, Marks G (2009). A postfunctionalist theory of European integration: From permissive consensus to constraining dissensus. British Journal of Political Science.

[CR67] Hooghe L, Marks G (2015). Delegation and pooling in international organizations. Review of International Organizations.

[CR68] Hooghe L, Marks G (2018). Cleavage theory meets Europe’s crises: Lipset, Rokkan, and the transnational cleavage. Journal of European Public Policy.

[CR69] Hooghe L, Marks G (2019). Grand theories on European integration in the 21^st^ century. Journal of European Public Policy.

[CR70] Hooghe, L., & Marks, G. (2022). *The social roots of the transnational cleavage: Sex, education, and occupation*. European University Institute, RSC Working Paper 2022/57. 10.2139/ssrn.4171743

[CR71] Hooghe, L., Marks, G., Lenz, T., Bezuijen, J., Ceka, B., & Derderyan, S. (2017). *Measuring international authority: A postfunctionalist theory of governance*. Oxford University Press.

[CR72] Hooghe, L., Lenz, T., & Marks, G. (2019a). *A theory of international organization*. Oxford University Press.

[CR73] Hooghe L, Lenz T, Marks G (2019). Contested world order: The delegitimation of global governance. Review of International Organizations.

[CR74] Hopf T (2010). The logic of habit in international relations. European Journal of International Relations.

[CR75] Hurrelmann, A., & Schneider, S. (eds.). (2015). *The legitimacy of regional integration in Europe and the Americas*. Palgrave Macmillan.

[CR76] Hutter, S., Grande, E., & Kriesi, H. (2016). *Politicizing Europe: Integration and mass politics*. Cambridge University Press.

[CR77] Johnson T (2013). Institutional design and bureaucrats' impact on political control. Journal of Politics.

[CR78] Johnson T, Urpeleinen J (2014). International bureaucrats and the formation of intergovernmental organizations: Institutional design discretion sweetens the pot. International Organization.

[CR79] Jupille, J., Mattli, W., & Snidal, D. (2013). *Institutional choice and global commerce*. Cambridge University Press.

[CR80] Keohane, R. (1984). *After hegemony: Cooperation and discord in the world political economy*. Princeton University Press.

[CR81] Keohane, R., & Hoffmann, S. (1991). Institutional change in Europe in the 1980s. In Keohane, R. & Hoffmann, S. (eds.), *The new European Community: Decision-making and institutional change* (pp. 1–39). Westview. 10.1007/978-1-349-23984-9_21

[CR82] Kindleberger C (1973). The world in depression.

[CR83] Kono D (2007). Making anarchy work: International legal institutions and trade cooperation. Journal of Politics.

[CR84] Koremenos B (2005). Contracting around international uncertainty. American Political Science Review.

[CR85] Koremenos, B. (2008). When, what, and why do states choose to delegate? *Law and Contemporary Problems, 71*(1), 151–192. https://www.jstor.org/stable/27592225

[CR86] Koremenos, B. (2016). *The continent of international law: Explaining agreement design*. Cambridge University Press.

[CR87] Koremenos B, Lipson C, Snidal D (2001). The rational design of international institutions. International Organization.

[CR88] Krasner SD (1984). Approaches to the state: Alternative conceptions and historical dynamics. Comparative Politics.

[CR89] Kriesi, H., Grande, E., Lachat, R., Dolezal, M., Bornschier, S., & Frey, T. (2008). *West European politics in the age of globalization*. Cambridge University Press.

[CR90] Lake, D. (2007). Delegating divisible sovereignty: Sweeping a conceptual minefield. *Review of International Organizations,**2*(3), 219–237. 10.1007/s11558-007-9012-3

[CR91] Lazer D (2011). Networks in political science: Back to the future. PS-Political Science and Politics.

[CR92] Lenz T, Burilkov A, Viola L (2019). Legitimacy and the cognitive sources of international institutional change: The case of regional parliamentarization. International Studies Quarterly.

[CR93] Lipscy, P. (2017). *Renegotiating the world order: Institutional change in international relations*. Cambridge University Press.

[CR94] Lührmann A, Tannenberg M, Lindberg SI (2018). Regimes of the world (RoW): Opening new avenues for the comparative study of political regimes. Politics and Governance.

[CR95] Mahoney, J., & Thelen, K. (2010). A theory of gradual institutional change. In Mahoney, J. & Thelen, K. (eds.), *Explaining institutional change: Ambiguity, agency, and power* (pp. 1–37). Cambridge University Press. 10.1017/CBO9780511806414.003

[CR96] Mansfield E, Milner HV (2012). Votes, vetoes, and the political economy of international trade agreements.

[CR97] Mansfield E, Milner H, Rosendorff P (2002). Why democracies cooperate more: Electoral control and international trade agreements. International Organization.

[CR98] Mansfield E, Milner HV, Pevehouse JC (2008). Democracy, veto players and the depth of regional integration. The World Economy.

[CR99] Marks, G., Lenz, T., Ceka, B., & Burgoon, B. (2014). Discovering cooperation: A contractual approach to institutional change in regional international organizations. Robert Schumann Center for Advanced Studies (RSCAS) Working Paper Series, Nr. 2014/65, Florence: European University Institute. 10.2139/ssrn.2446441

[CR100] Martin, L. (1992). Interests, power and multilateralism. *International Organization,**46*(4), 765–792. 10.1017/S0020818300033245

[CR101] Mattli, W. (1999). *The logic of regional integration: Europe and beyond*. Cambridge University Press.

[CR102] Menem, C. (1996). *¿Qué es el Mercosur?* Ediciones Ciudad Argentina.

[CR103] Mercosur. (1991). *Treaty for the establishment of a common market between Argentina, Brazil, Paraguay and Uruguay*. Asunción, March 26.

[CR104] Moe T (2012). Delegation, control, and the study of public bureaucracy. The Forum.

[CR105] Nelson, S., & Weaver, C. (2016). Organizational culture. In Cogan, J.K., Hurd, I. & Johnstone, I. (eds.), *Oxford handbook of international organizations* (pp. 920–939). Oxford University Press. 10.1093/law/9780199672202.003.0043

[CR106] OECS. (1968). *Agreement establishing the East Caribbean Common Market*. Grenada, June 11.

[CR107] Owen E, Johnston NP (2017). Occupation and the political economy: Job routineness, offshorability, and protectionist sentiment. International Organization.

[CR108] Pierson P (1996). The path to European integration: A historical institutionalist analysis. Comparative Political Studies.

[CR109] Pierson P (2000). The limits of design: Explaining institutional origins and change. Governance.

[CR110] Pierson, P. (2004). *Politics in time: History, institutions, and social analysis*. Princeton University Press.

[CR111] Pollack, M. A. (2003). *The engines of European integration: Delegation, agency, and agenda setting in the EU*. Oxford University Press.

[CR112] Powers K, Goertz G (2011). The economic-institutional construction of regions: Conceptualization and operationalization. Review of International Studies.

[CR113] Ribeiro Hoffmann, A. (2015). Politicization and legitimacy in Mercosur. In Hurrelmann, A. & Schneider, S. (eds.), *The legitimacy of regional integration in Europe and the Americas* (pp. 57–73). Palgrave Macmillan. 10.1057/9781137457004_4

[CR114] Riggirozzi, P. (2015). The social turn and contentious politics in Latin American post-neoliberal regionalism. In Hurrelmann, A. & Schneider, S. (eds.), *The legitimacy of regional integration in Europe and the Americas* (pp. 229–248). Palgrave Macmillan. 10.1057/9781137457004_13

[CR115] Rixen T, Viola LA (2014). Putting path dependence in its place: Toward a taxonomy of institutional change. Journal of Theoretical Politics.

[CR116] Rocabert, J., Schimmelfennig, F., Crasnic, L., & Winzen, T. (2019). The rise of international parliamentary institutions: Purpose and legitimation. *Review of International Organizations,**14*(4), 607–631. 10.1093/oso/9780198864974.003.0002

[CR117] Rosenbaum PR (2005). Heterogeneity and causality: Unit heterogeneity and design sensitivity in observational studies. American Statistician.

[CR118] Scharpf, F. W. (1999). *Governing in Europe: Effective and democratic?* Oxford University Press.

[CR119] Schimmelfennig F (2018). European integration (theory) in times of crises: A comparison of the Euro and Schengen crises. Journal of European Public Policy.

[CR120] Schmitter P (1969). Three neo-functional hypotheses about international integration. International Organization.

[CR121] Schmitter P (1970). A revised theory of regional integration. International Organization.

[CR122] Scipioni M (2018). Failing forward in EU migration policy? EU integration after the 2015 asylum and migration crisis. Journal of European Public Policy.

[CR123] Sekhon, J. S. (2008). The Neyman-Rubin model of causal inference and estimation via matching methods. In Box-Steffensmeier, J., Brady, H. & Collier, D. (eds.), *Oxford handbook of political methodology* (pp. 271–299). Oxford University Press. 10.1093/oxfordhb/9780199286546.003.0011

[CR124] Simmons, B. (2009). *Mobilizing for human rights: International law in domestic politics*. Cambridge University Press.

[CR125] Singer JD (1988). Reconstructing the Correlates of War dataset on material capabilities of states, 1816–1985. International Interactions.

[CR126] Sommerer, T., Squatrito, T., Tallberg, J., & Lundgren, M. (2022). Decision-making in international organizations: Institutional design and performance. *Review of International Organizations,**17*(4), 815–845. 10.1007/s11558-021-09445-x 10.1007/s11558-023-09492-6 PMC10242228 37360543

[CR127] Stier S (2015). Democracy, autocracy and the news: The impact of regime type on media freedom. Democratization.

[CR128] Stone, R. (2011). *Controlling institutions: International organizations and the global economy*. Cambridge University Press.

[CR129] Stone Sweet A, Brunell T (1998). Constructing a suprantional constitution: Dispute resolution and governance in the European Community. American Political Science Review.

[CR130] Stone Sweet A, Sandholtz W (1997). European integration and supranational governance. Journal of European Public Policy.

[CR131] Streeck, W., & Thelen, K. (eds.). (2005). *Beyond continuity: Institutional change in advanced political economies*. Oxford University Press.

[CR132] Tallberg J, Zürn M (2019). The legitimacy and legitimation of international organizations: Introduction and framework. Review of International Organizations.

[CR133] Tallberg, J., Sommerer, T., Squatrito, T., & Jönsson, C. (2013). *The opening up of international organizations: Transnational access in global governance*. Cambridge University Press.

[CR134] Tallberg J, Sommerer T, Squatrito T, Jönsson C (2014). Explaining the transnational design of international organizations. International Organization.

[CR135] Tallberg J, Sommerer T, Squatrito T (2016). Democratic memberships in international organizations: Sources of institutional design. Review of International Organizations.

[CR136] Tarrow, S. (2005). *The new transnational activism*. Cambridge University Press.

[CR137] Thompson, J. D. (2003). *Organizations in action*. Transaction Publishers.

[CR138] Tsebelis, G. (2002). *Veto players: How political institutions work*. Princeton University Press.

[CR139] Voeten E (2019). Making sense of the design of international institutions. Annual Review of Political Science.

[CR140] Voeten, E. (2021). *Ideology and international institutions*. Princeton University Press.

[CR141] Voeten, E. (2022). Is the public backlash against globalization a backlash against legalization and judicialization? *International Studies Review,**22*(2), 1–17. 10.1146/annurev-polisci-041719-102405

[CR142] Walter S (2021). The backlash against globalization. Annual Review of Political Science.

[CR143] Wendt A (2001). Driving with the rearview mirror: On the rational science of institutional design. International Organization.

[CR144] Zürn, M. (2018). *A theory of global governance: Authority, legitimacy and contestation*. Oxford University Press.

[CR145] Zürn M, Binder M, Ecker-Ehrhardt M (2012). International authority and its politicization. International Theory.

