# Supplementary material for: Discovering cooperation: Endogenous change in international organizations
Source: Rev Int Organ. 2022 Dec 14:1–36. Online ahead of print. doi: 10.1007/s11558-022-09482-0 (PMC9748392; doi:10.1007/s11558-022-09482-0)
Supplement: Supplementary file 1 — Supplementary file1 (PDF 774 KB) [file 11558_2022_9482_MOESM1_ESM.pdf]

Appendix A: Sample of international organizations, first year-last year in dataset

Africa (10)
African Union (AU), 1963-2019
Economic and Monetary Community of Central Africa (CEMAC), 1973-2019
Economic Community of Western African States (ECOWAS), 1975-2019
Common Market for Eastern and Southern Africa (COMESA), 1986-2019
East African Community I (EAC1), 1967-1976
East African Community II (EAC2), 1993-2019
Economic Community of Central African States (ECCAS), 1985-2019
Intergovernmental Authority on Development (IGAD), 1986-2019
Southern African Customs Union (SACU), 1969-2019
Southern African Development Community (SADC), 1981-2019

Americas (9)
Latin American Integration System (ALADI), 1961-2019
Andean Community (CAN), 1969-2019
Caribbean Community (Caricom), 1968-2019
Common Market of the South (Mercosur), 1991-2019
North American Free Trade Association (NAFTA), 1994-2019
Organization of American States (OAS), 1951-2019
Organization of Eastern Caribbean States (OECS), 1968-2019
Latin American and Caribbean Economic System (SELA), 1976-2019
Central American Integration System (SICA), 1952-2019

Asia-Pacific (6)
Asia-Pacific Economic Cooperation (APEC), 1991-2019
Association of Southeast Asian Nations (ASEAN), 1967-2019
Pacific Islands Forum (PIF), 1973-2019
Shanghai Cooperation Organization (SCO), 2002-2019
South Asian Association for Regional Cooperation (SAARC), 1986-2019
South Pacific Community (SPC), 1950-2019

Europe (12)
Benelux Community (Benelux), 1950-2019
Central Commission for the Navigation of the Rhine (CCNR), 1950-2019
European Centre for Nuclear Research (CERN), 1954-2019
Commonwealth of Independent States (CIS), 1992-2019
Council for Mutual Economic Assistance (COMECON), 1959-1991
Council of Europe (COE), 1950-2019

European Economic Area (EEA), 1994-2019
European Free Trade Association (EFTA), 1960-2019
European Space Agency (ESA), 1980-2019
European Union (EU), 1952-2019[1]
Nordic Council (Nordic), 1952-2019
Organization for Security and Cooperation in Europe (OSCE), 1973-2019

Middle East and North Africa (4)
Arab Maghreb Union (AMU), 1989-2019
Gulf Cooperation Council (GCC), 1981-2019
League of Arab States (LOAS), 1945-2019
Organization of Arab Petroleum Exporting States (OAPEC), 1968-2019

[1] We conceive the European Coal and Steel Community Treaty as the first treaty on European Union, but an analysis that conceives the ECSC and the post-1957 European Union as separate organizations produces the same results.

Appendix B: Description of dependent variable *Delegation*

This section draws on excerpts from the MIA measure for delegation conceptualized and operationalized in Hooghe, Marks, Lenz, Bezuijen, Ceka, and Derderyan (2017, pp. 107–113).

Delegation is a conditional grant of authority by member states to an independent body, such as a general secretariat that can set the agenda for decision making, an executive that takes day-to-day decisions, or a court that can impose a sanction on a non-compliant state. The delegate—in this case, the non-state actor—gains some influence over decision making; the principals—the member states—gain a capacity for governance that does not depend on their active presence.

The dependent variable *Delegation* is an annual measure of the allocation of authoritative competences to non-state bodies in an IO's decision-making process (Hooghe et al. 2017: 107-113). A distinction is made between political delegation and judicial delegation. Political delegation in setting the IO agenda or taking the final IO decision is assessed:

- in one or more IO bodies (assemblies, executives, general secretariats, consultative bodies) that are
- partially or fully, composed of non-member-state actors, which
- exercise or co-exercise authority over agenda setting or final decision making
- in one or more of six decision areas: membership accession, membership suspension, constitutional reform, budgetary allocation, financial non-compliance, and up to five streams of policy making.

Judicial delegation is the conditional transfer of authority to courts, arbitrators, or tribunals. It is assessed with items that tap how obligatory and independent legal dispute settlement is, how binding, whether there is a standing tribunal, who has access, whether there is a remedy for non-compliance, and whether it can make compulsory preliminary rulings.

The scoring for delegation works as follows:

1. Each body receives a composition score for the degree to which it is non-state. All scores range from 0 to 1.
2. Composition scores for all bodies that participate in agenda setting are averaged in each decision area after two adjustments. An adjustment is made for a general secretariat that gatekeeps agenda setting, and an adjustment is made when an IO has more than one policy stream. This produces an agenda setting score for each decision area.
3. We identify the body with the highest (i.e. most non-state) composition score in final decision making in each decision area. This is the final decision score for each decision area.
4. A dispute settlement score is calculated for each decision area.
5. We now have three scores for each decision area: an agenda setting score, a final decision score, and a dispute settlement score. The average of these scores is the delegation score

for a decision area. The delegation score for an IO is the average of the delegation scores across the six decision areas.

Composition Scores

The first step in estimating delegation is to assess the extent to which an IO body is composed of non-state actors.

An IO body may be partially or fully independent of member states in one of three ways. It may be composed of representatives of bodies outside the executive organs of the member state, for example, representatives of national or regional parliaments, courts, interest groups, professional associations, or international organizations.[2] Or it may be composed of one or more members of an IO body who operate under an explicit norm of independence from member state control. Or it may be an external non-state body, such as an international organization that plays an independent decision-making role in a second international organization. In each case, the participant in an IO body must have full voting rights to qualify as non-state.

GENERAL SECRETARIAT

A general secretariat receives a composition score of 1 when it consists of a permanent core of non-state actors with at least one of the following properties: the officials of the secretariat have international diplomatic status; they are required to take an oath of independence; member states are required to refrain from influencing the general secretariat. An IO administration receives a score of zero if none of the above conditions is met and/or the administration is lodged in one or more member state administrations or rotates among them.

ASSEMBLY

Most IOs have member state-dominated assemblies, but some have independent assemblies in which some or all members are popularly elected or are selected by national parliaments, regional governments, local governments, trade unions, business associations, or other non-state groups. Each assembly present in an IO is scaled as follows, with the applicable composition score in brackets:

How are members of the assembly selected?

- All members selected by member states (0)
- A majority, but not all, selected by member states (0.33)
- At least 50 percent of the members of the assembly are selected by parliaments, subnational governments, or other non-member state actors (0.66)
- At least 50 percent of the members of the assembly are popularly elected (1)

EXECUTIVE

The composition of an executive is non-state when those who sit and vote in an executive do not receive voting instructions from their government.

[2] A national executive is defined to include ministers of the central government, diplomats, military or security attachés, central bankers, civil servants, and experts representing their national government.

This is assessed by examining the explicit constraints on member state command in relation to some proportion of the members of the executive. For example, one or more members of the executive may be required to take an oath of independence or may be constitutionally bound to act on behalf of the organization rather than his or her member state. We scale each executive in an IO as follows:

Do members of the executive directly represent member states?

- All members receive voting instructions from a government (0)
- 50 percent or more, but not all, members receive voting instructions
- from a government (0.33)
- Fewer than 50 percent of the members receive voting instructions from a government (1)

OTHER IO BODIES

Member states receive a compositional score of zero where they play an individual role in agenda setting or the final decision. International organizations that play a role in agenda setting or the final decision of another IO receive a score of 1. Consultative bodies, that is, bodies composed of non-state representatives selected by national or subnational assemblies, representatives of business, trade unions, social movements, or professional experts, have a composition score of 1.

Delegation in Agenda Setting and the Final Decision

The next step is to identify those bodies that take part in agenda setting and the final decision in each decision area. Each body has a separate column in the dataset with a value—its composition score—in the row indicating the decision stage at which it participates. For the sample of forty-six IOs in the period 1950–2019, this requires fourteen columns: three columns each for assemblies, executives, and consultative bodies; two columns for general secretariats; one column for the dispute settlement body; one column for individual member states; and one column for a non-state actor not captured by the preceding options (e.g. an international organization that operates as a non-state decision maker in this IO).

Next, for agenda setting, we ask who can initiate a) the accession of new members; b) the suspension of a member state; c) constitutional reform; d) drafting the budget; e) proceedings on financial compliance; f) policy (up to five policy streams). To assess who is involved in the final decision, the same six decision areas are evaluated.

AGGREGATE DELEGATION IN AGENDA SETTING

Composition scores for delegation for all IO bodies involved in agenda setting in each of the six decision areas are averaged. When an IO has more than one policy stream, the composition scores are averaged across the policy streams to produce an aggregate policy stream score. This aggregate score is then used as the policy stream score when we average across the six decision areas.

The aggregate score for delegation in agenda setting for an IO is the average score for accession, suspension, constitutional reform, budget, financial compliance, and policy making. This score, like every one of its components, ranges from 0 to 1.

AGGREGATE DELEGATION IN THE FINAL DECISION

The same composition scores are used to calculate an aggregate score for delegation in the final decision. Rather than averaging scores, we assess whether a body composed to some degree by non-state actors is in a position to block a decision. Whereas we estimate delegation in agenda setting by identifying all bodies that are involved in agenda setting, we ask instead whether the final decision runs through a non-state body, and if so, how non-state is its composition. So we first identify the most non-state actor in each decision area, allocate the appropriate composition score to that body, and then average across decision areas. This score varies between 0 and 1, as do all its components.

Delegation in Dispute Settlement

Legal or judicial dispute settlement is the third and final component of the delegation measure. The measure of dispute settlement is concerned with arbitration and adjudication. It excludes diplomatic or political forms of dispute settlement involving negotiation, mediation, or conciliation by a third party which, if routinized in an IO body and involving non-state actors, are encompassed in the measure as political delegation.

The score for dispute settlement is the average of seven components scaled from 0 to 1. If an IO has two dispute settlement mechanisms, the final score of the most supranational mechanism is used. The items are as follows with scores in brackets.

Third party dispute settlement is estimated along seven dimensions. Each component is scaled from zero to one.

- Is there a dispute settlement system; can member states opt out; or is it obligatory for all member states (0, 0.5, 1)?
- Is there a right for third-party review of disputes; is this right mediated by a political body; or is it an automatic right (0, 0.5, 1)?
- Is there a tribunal; is its composition ad hoc; or is it a standing tribunal (0, 0.5, 1)?
- Are rulings non-binding; conditionally binding; or binding (0, 0.5, 1)?
- Who has access to dispute settlement: member states only; the general secretariat; non-state actors as well as states (0, 0.5, 1)?
- Is there no remedy; partial remedy (retaliatory sanctions); or full remedy (direct effect) (0, 0.5, 1)?
- Is there a preliminary ruling system; is it voluntary; or is it compulsory (0, 0.5, 1)?

Aggregate Delegation Scores

The variable, Delegation, is the unweighted average of delegation in agenda setting, delegation in final decision, and judicial delegation across six decision areas: accession, suspension, constitutional reform, budgetary allocation, financial compliance, and policy making.

Estimation routine

Two Ph.D students and one postdoctoral fellow implemented the MIA coding scheme on 41 IOs for 2010-2019. They worked under close supervision of two faculty, one of whom had extensive prior experience in using the MIA scheme for coding the authority of international organizations. Before starting the coding process, coders were first instructed to make themselves familiar with the IO profile, which documents each coding decision for up to 2010 and provides references. This information is in the public domain (Hooghe et al. 2017: 167-668; https://garymarks.web.unc.edu/data/international-authority/). Next, they were instructed to update this information for the additional years after 2010, and to do this by pooling their efforts in information gathering and interpretation. Hence, estimates for each IO-year-dimension were derived through sustained, open-ended discussion among coders rather than by averaging independent coder decisions, in an effort to privilege validity over intercoder reliability. Coders consulted the detailed manual "How to apply the coding scheme" developed by the original MIA team to inform decisions on ambiguous or borderline cases (Hooghe et al. 2017: 34-106).

Appendix C: Description of independent variables

This appendix describes the independent variables used in our analyses, starting with the three core variables of interest.

Contract

Contract is a dichotomous variable. A contract is *closed-ended* (value=0) if its purpose is to achieve a fixed objective of interstate cooperation under clearly specified conditions. Closed-ended contracts identify the means to achieve cooperation in codified policy areas. A contract is *open-ended* (value=1) if its purpose is to attain broad-ranging cooperation among governments or peoples under weakly specified conditions. Open-ended contracts focus on the process because the outcome is indeterminate, that is to say, they are expressed in language that avoids specifying the end-destination. Examples illustrate the relevant categories of contractual incompleteness.

All free trade agreements are closed contracts: the contracting parties are strictly governments, the contract has a circumscribed objective of eliminating barriers to trade, and the end-destination is set to establish a free trade zone. For example, the Dickinson Bay agreement establishing the Caribbean Free Trade Organization (CARIFTA) restricts cooperation to free trade: "AWARE that the broadening of domestic markets through the elimination of barriers to trade between the territories is a prerequisite to [full employment and improved living standards]; CONVINCED that such elimination of barriers to trade can best be achieved by the immediate establishment of a Free Trade Economic Community for all the countries who so desire" (Preamble). The Latin American Free Trade Association also articulates a specific goal: "By the present Treaty, the Contracting Parties establish a free-trade-zone" (Art. 1, 1960 Montevideo Treaty). It delineates a program of trade liberalization based on periodic negotiations between member states, the removal of tariffs based on national and common lists, and details flanking measures in industry, tax policy and agriculture.

IOs that organize collaboration in a sector or policy problem tend to have a closed-ended contract. The objective of the Organization of Arab Petroleum Export Countries (OAPEC) is clearly specified: "The principal aim of the Organization shall be the co-ordination and unification of the petroleum policies of Member Countries" (1968 OAPEC Agreement), and its rules and regulations are designed to cover all exigencies.

Open-ended contracts commit to broad-ranging cooperation expressed in language that avoids specifying the end-destination of cooperation. Most economic unions fall in this category. An economic union is typically more multi-purpose than a free trade agreement, both with regard to its objectives and its means; it also tends to describe these goals in language that leaves the end-destination unclear – unlike for a free trade agreement. A good example is Benelux 2.0, which had as its central goal to establish an economic union with "the principal aim" to achieve "economic progress" (1958 Treaty establishing the Benelux Economic Union, preamble). This is further translated into three broadly worded purposes: "to strengthen the economic ties between their countries by means of free movement of persons, goods, capital and services;" "to co-ordinate their policies in the economic, financial and social fields in order to attain the most satisfactory level of employment and the highest standard of living;" "to pursue a joint trade policy . . . by means of the free-est possible trade."

The Shanghai Cooperation Organization (SCO) has broad-ranging goals that range from the purpose to "facilitate comprehensive and balanced economic growth, social and cultural development in the region through joint action on the basis of equal partnership" to "consolidate multidisciplinary cooperation in the maintenance and strengthening of peace, security and stability in the region" (SCO Charter, Art. 1).

The most open-ended contracts engage peoples as well as governments in an open-ended venture. They commit states to a vague purpose—e.g. a "community of peoples" or "ever closer union"—to be achieved by unspecified actors through an open-ended process. Cooperation is framed as an evolutionary process that is revealed only over time. The idiomatic case is the European Union along with its predecessors. Consistently from the 1951 ECSC Treaty through the 2009 Lisbon Treaty, the preamble to the EU contained very broad language even while the wording has changed. The preamble of the ECSC Treaty reads as follows: "RESOLVED to substitute for historic rivalries a fusion of their essential interests; to establish, by creating an economic community, the foundation of a broad and independent community among peoples long divided by bloody conflicts; and to lay the bases of institutions capable of giving direction to their future common destiny." The latter phrase was refined to "an ever closer union," which entered the contract in the 1957 Rome Treaty of the European Economic Community. The wording in the 2009 Lisbon Treaty is as follows: "RESOLVED to continue the process of creating an ever closer union among the peoples of Europe, in which decisions are taken as closely as possible to the citizen in accordance with the principle of subsidiarity."

To evaluate whether an IO has a closed or an open-ended contract, we use a lexicon of key words to the preamble and assess the paragraphs stating intent of the foundational treaties and their updates. We applied the coding schema initially to the foundational treaties of 35 regional organizations (Marks et al. 2014). We then compared these scores with those of two independent researchers familiar with the study's concepts who each coded thirteen randomly chosen regional organizations. They agreed on all but one score, producing a Krippendorff's alpha of 0.78.[3] In the next stage, we implemented the coding, using the same lexicon, to the remaining IOs, and we assessed the extent of open-endedness of all foundational treaties over an IO's lifetime.

Lexical key words include the following:

- Union of peoples
- Ever closer union
- Homogenous society
- Community of peoples
- Shared destiny
- Political federation
- Aspiring to achieve the integration of [geographic region]; and
- Other phrases that suggest open-ended cooperation/integration

[3] Krippendorff's alpha measures agreement among coders and ranges from zero, which indicates no agreement beyond chance, to one, which indicates agreement without exceptions.

Policy Scope

We measure policy scope (or policy portfolio) as the number of policies in which an IO is engaged in a given year across a list of twenty-five policies (see below). This is more fine-grained than classifications of IO mandate, such as the three-way distinction between economic, security, and multi-issue IOs in the Correlates of War dataset (Boehmer et al. 2004). It is also more fine-grained, covers more IOs, and includes more policies than measures developed for regional organizations (Balassa 1961; Haftel 2013), security IOs (Haftel and Hofmann 2017), or IO policy output (Lundgren et al. 2018). In constructing a dictionary for policy categories, we draw from extant policy dictionaries (such as the Comparative Agenda project), case studies of international organizations and agreements, and IO documentation.

List of policy categories

1.	Agriculture
2.	Competition policy, mergers, state aid, antitrust
3.	Culture and media
4.	Education (primary, secondary, tertiary), vocational training, youth
5.	Development, aid to poor countries
6.	Financial regulation, banking regulation, monetary policy, currency
7.	Welfare state services, employment policy, social affairs, pension systems
8.	Energy (coal, oil, nuclear, wind, solar)
9.	Environment: pollution, natural habitat, endangered species
10.	Financial stabilization, lending to countries in difficulty
11.	Foreign policy, diplomacy, political cooperation
12.	Fisheries and maritime affairs
13.	Health: public health, food safety, nutrition
14.	Humanitarian aid (natural or man-made disasters)
15.	Human rights: social & labor rights, democracy, rule of law, non-discrimination, election monitoring
16.	Industrial policy (including manufacturing, SMEs, tourism)
17.	Justice, home affairs, interior security, police, anti-terrorism
18.	Migration, immigration, asylum, refugees
19.	Military cooperation, defense, military security
20.	Regional policy, regional development, poverty reduction
21.	Research policy, research programming, science
22.	Taxation, fiscal policy coordination, macro-economic policy coordination
23.	Telecommunications, internet, postal services
24.	Trade, customs, tariffs, intellectual property rights/patents
25.	Transport: railways, air traffic, shipping, roads

To be categorized as an IO policy, a policy needs to meet two general criteria. First, it is a multilateral policy administered by the IO rather than an aggregation of bilateral policies among the member states. ASEAN provides an example. From 2000, ASEAN countries began to coordinate their management of regional short-term liquidity problems by setting up bilateral swap arrangements—the so-called Chian Mai Initiative, but this does not meet the multilateral criterion. This becomes multilateral from March 2010, when the Chiang Mai Initiative

Multilateralization (CMIM) Agreement authorized ASEAN to administer management of regional short-term liquidity problems, and we begin categorizing "Financial stabilization and lending to countries in difficulty" as an ASEAN policy.

A second criterion is that the policy is institutionalized. This requires a tangible legal, financial, or organizational footprint – not merely declarations of intent – evidenced in documentation, e.g. treaties, protocols, declarations, constitutions, framework legislation, budgetary documents, or white papers.

The following eight indicators tap whether there is tangible evidence that an IO's portfolio encompasses a particular policy:

- The policy features in the name of the organization;
- The policy is highlighted as a central purpose of the IO in the opening paragraphs of its foundational contract;
- The policy is the primary subject of a separate treaty section;
- The policy is the primary subject of an annex, a protocol, a convention, an agreement;
- The policy is explicitly tied to budgetary resources in a convention, constitution, protocol, annexes, or ancillary document;
- The policy is the primary subject of an (actually existing) IO instrument: agency, fund, directorate, or tribunal;
- The policy is the primary subject of an (actually existing) IO intergovernmental committee, council, working group or equivalent;
- The policy features as the functional specialization of the national representatives who sign the IO's foundational document.

Policy scope is assessed at each reform moment of an IO, i.e. at the time of treaty revision, new protocol or convention, the passing of framework legislation, or the creation of a new IO body or instrument. For recent decades in particular, one can often find valuable information on the IO's website, from NGOs, and from academics monitoring an IO's activity.

Politicization

Politicization estimates the salience and divisiveness of debate over an IO. Our measure estimates media coverage of protests directed at an IO on the grounds that protests that reverberate in the news will intensify concerns about an IO's legitimacy and may motivate decision makers to adjust IO design. We adapt a measure developed by Tallberg et al. (2014), which captures annual media coverage of protests or demonstrations directed at an IO in the world's leading newspapers. Like them, we use the Lexis-Nexis database.

The estimate is the number of articles that combine "PROTESTOR" or "DEMONSTRATOR" with the IO name at least once per article (articles with multiple mentions are only counted once). Variations of "protestor" and "demonstrator" are used to pick up strings such as "protest" and "demonstration." We use a three-year moving average—the equally weighted moving average of politicization at t, $t-1$, and $t-2$—to reflect the notion that the pressure for a response is strongest in the year of the protest and the first few years after the protest, but then recedes quickly. This moving average is divided by 100 to get more readable numbers.

Our baseline search segment, created in the “Build Your Own Segment Search”, reads as follows:

“organization name” OR “organization acronym” w/p demonstrator OR protestor OR protester OR protest OR demonstration

For organizations with important component institutions, we modify the baseline search segment to include these institutions. This is only done when the component institution does not contain a segment that is the organization’s name. The following are instances in which we modify the search terms, along with the search terms used (*w/p demonstrator OR protestor OR protester OR protest OR demonstration* omitted):

- **European Union**: *"European Commission" OR "European Parliament" OR "Council of the European Union" OR "European Council" OR "European Coal and Steel Community" OR “ECSC” OR "European Economic Community" OR "European Community" OR "European Union" OR “EEC” OR “ECJ” OR "European Court of Justice" OR “CJEU” OR "Court of Justice of the European Union" OR "Court of Justice of the European Communities" OR "European Central Bank" OR “ECB” OR “CJEC” OR "European Court of Auditors" OR "European Investment Bank"*
- **African Union**: *"African Court on Human and Peoples' Rights" OR "African Court of Human Rights" OR "African Union" OR "Organisation of African Unity" OR "Organization of African Unity" OR “OAU” OR “AU” OR " African Court of Justice and Human Rights"*
- **Council of Europe**: *"Council of Europe" OR "European Court of Human Rights" OR "Venice Commission" OR "Committee of Ministers" OR “ECtHR” OR “ECHR” OR “CoE”*

Some organizations have acronyms that can also refer to something other than the organization. We deal with this in two ways. For some organizations, generally those with high numbers of newspaper articles, we exclude the acronym. These include:

- The Andean Community’s Spanish acronym, CAN, is too general, so we search only *“Andean Community” OR “Comunidad Andina.*”
- The Nordic Council’s acronym, Nordic, is too general, so we search only “*Nordic Council.*”

For other organizations, generally those with lower numbers of newspaper articles, we keep the acronym, and check each article and exclude those that refer to something other than the organization. These include the following (we also mention the confounding double use):

- SACU refers also to a cricket union
- SPC refers also to a military title abbreviation
- SELA refers also to a surname
- AMU refers also to first or surnames
- CoE refers often to a surname. In this case, we search the articles that emerge from the original search for “CoE,” then check each one of these articles.

Other organizations pose challenges for diverse reasons. Since most of these organizations generally return lower numbers of newspaper articles, we maintain the baseline

search segment, check each article and exclude those that refer to something other than the organization.

- Using demonstrator in the search term for the ESA is problematic because it refers to a technical space-specific term.
- Benelux may refer to the Benelux countries rather than the organization.

Each result of a search query is verified for validity by examining the headline and body of the article. We search by year using LexisNexis' default "Major World Publications" filter, which can be found under the "Source Type" section of Advanced Options. We search as far back as organizations go in our dataset, plus a two-year lag. However, we note that LexisNexis coverage varies across several dimensions. For one, some major publications are not included prior to the 1980s.[4] Furthermore, the composition of the "Major World Publications" filter changes over time as LexisNexis undergoes updates to its database of articles and sources, as do the articles that are returned by the search queries outlined in this section. Therefore, our measure represents a snapshot of LexisNexis at the time of data collection (January 2021). Although similar to previous measures of politicization that covered the 1950-2010 period, it is not a perfect replication due to the circumstances outlined above.

The 2010-2019 period displays two dynamics that lead to growing rates of politicization for all organizations as compared to 1950-2010. First, on a general level, the 24/7 news cycle, increasing online presence of legacy news organizations, and growing number of media in general lead to higher rates of reporting for all events. This is particularly prominent for dramatic events like the Arab Spring or the 2015 migrant crisis in Europe, each of which led to thousands of hits for the relevant organizations. Second, this higher rate of reporting is coupled with the dramatic events of the period, notably the Arab Spring, annexation of Crimea, and migrant crisis in Europe, all of which have led to politicization rates for relevant organizations to be orders of magnitude greater than the mean.

Historical ties

This is a dichotomous variable that takes on a value of 1 if two-thirds of an IO's founding members (1) share a history of membership within a federation, or (2) share experience of membership within—and resistance to—a colonial empire. This shared history meets the following criteria: the political (con)federation or colonial empire endured for at least twenty years, and it was in existence no more than fifty years prior to the creation of the IO. The table below lists the IOs in our sample that meet these criteria. *Source*: Hooghe, Lenz, Marks (2019).

[4] Three major publications illustrate this: coverage of the Guardian extends as far back as 1975, the Financial Times as far back as 1982, and the New York Times as far back as 1980.

Historical ties among IO founding members

IO name	End of ties	IO creation	Description of historical ties
Arab Maghreb Union (AMU)	±1960	1989	4 of 5 founding members are former French colonies
Benelux	1839<br>ongoing	1944	federation between 1815-39;<br>BLEU: Belgium-Lux economic & monetary union (from 1922)
Caribbean Community (CARICOM)	1962	1968	former British colonies; former West Indies Federation (1958-62)
Central American Integration System (SICA)	1922	1952	Federal republic of Central America (1823-41); five short-lived attempts, most recently the Federation of Central America (1921-22)
Commonwealth of Independent states (CIS)	1991	1992	former members of the Soviet Union federation
Common Market for Eastern and Southern Africa (COMESA)	±1960	1982	8 of 12 founding members are former British colonies
East African Community I (EAC 1)	1961-67	1967,<br>1993	former British colonies (until 1961); East African High Commission (EAHC) (1948-61); East African Common Services Organization (EACSO) (1961-67)
East African Community II (EAC 2)	1961-67	1993	see EAC 1
Economic and Monetary Union of Central African States (CEMAC)	1958	1966	former French colonies; Federation of Equatorial French Africa (AEF) (1910-1958)
Gulf Cooperation Council	1971	1981	4 of 6 founding members are former British colonies
Intergov. Authority on Development (IGAD)	±1960	1986	4 of 6 founding members are former British colonies
Nordic Council	1905	1952	colonial/confederal ties: Sweden-Finland (1150-1809); Norway-Denmark (1524-1814); Norway-Sweden (1814-1905); Denmark-Iceland (1524-1944)
Organization of Eastern Caribbean States (OECS)	1962	1968	former British colonies; former West Indies Federation (1958-62)
Pacific Islands Forum (PIF)	±1965	1973	5 of 7 founding members are former British colonies (2 other founding members are former colonies of New Zealand, itself founding member)

Trade interdependence

We use three different measures of trade interdependence that are widely applied in the literature: intra-IO trade share, trade intensity, and trade introversion. Intra-IO trade share is the simplest. It is the basic building block for the more compound other indices.

Bilateral trade data and data for some regional trade organizations are regularly published by international organizations. The most comprehensive data come from the UN COMTRADE

Database. Trade data before 1970 are spotty, so we only calculate statistics from 1970. We were able to use the algorithms made available by Philippe de Lombaerde's Institute on Comparative Regional Integration Studies the United Nations University in Bruges (UNU-CRIS). The website http://www.cris.unu.edu/riks/web/data is no longer live.

Intra-IO trade share is calculated using the following formula:

$$ITS_{i,t} = \frac{IT_{i,t}}{T_{i,t}} \times 100$$

where:
$IT_{i,t}$ denotes an IO's *i*'s intra-IO trade in year *t*,
$T_{i,t}$ denotes an IO *i*'s total trade in year *t* (*i*'s total imports plus total exports).

The value ranges from 0 to 100. This indicator reflects the importance of intra-IO trade (i.e. trade interdependence of member states) of a particular international organization in its overall trade.

Trade intensity relates intra-IO share to the size of world trade. In its simplest form, it is equal to the ratio of an IO's intra-IO trade share and its share of world trade. It is calculated using the following formula:

$$ITII_{i,t} = \frac{\left(\frac{IT_{i,t}}{T_{i,t}}\right)}{\left(\frac{T_{i,t}}{T_{w,t}}\right)}$$

where:
$IT_{i,t}$ denotes IO *i*'s intra-IO trade in year *t*,
$T_{i,t}$ denotes IO *i*'s total trade in year *t* (*i*'s total imports plus total exports),
$T_{w,t}$ denotes the world's total trade in year *t* (world's total imports plus total exports).

The value ranges from 0 to $\frac{T_{w,t}}{T_{i,t}}$.

This value is:

- equal to zero in the case of no intra-IO trade;
- equal to one if the organization's weight in its own trade is equal to its weight in world trade (geographic neutrality);
- higher than one if intra-IO trade is relatively more important than trade flows with the rest of the world;
- equal to the reciprocal of the organization's share in world trade when all trade is intra-IO (no extra-IO trade) – that is, the maximum value of the ITII index is the higher the smaller the organization's total trade.

Trade intensity's minimum value is 0, and there is no set maximum value.[5]

Trade introversion compares the relative size an IO's internal trade and external trade, and it rises (falls) only if the intensity of intra-IO trade grows more (less) rapidly than the intensity of extra-IO trade. It is defined as following:

$$STJ_{i,t} = \frac{\frac{HITI_{i,t}}{HETI_{i,t}} - 1}{\frac{HITI_{i,t}}{HETI_{i,t}} + 1} = \left(HITI_{i,t} - HETI_{i,t}\right) \Big/ \left(HITI_{i,t} + HETI_{i,t}\right)$$

with $HITI_{i,t}$ a homogeneous version of the intra-IO trade intensity index, the maximum value of which is independent from the IO *i*'s trade size. Its denominator is not the organization *i*'s share in world trade, but its share in the trade of the rest of the world:

$$HITI_{i,t} = \frac{\left(\frac{IT_{i,t}}{T_{i,t}}\right)}{\left(\frac{ET_{i,t}}{T_{w,t} - IT_{i,t}}\right)}$$

HETI (homogeneous extra-IO trade intensity index) is the complementary indicator of HITI. It is defined for IO *i* as:

$$HETI_{i,t} = \frac{1 - \left(\frac{IT_{i,t}}{T_{i,t}}\right)}{1 - \left(\frac{ET_{i,t}}{T_{w,t} - IT_{i,t}}\right)}$$

where:
$IT_{i,t}$ denotes organization *i*'s intra-IO trade in year *t*,
$ET_{i,t}$ denotes organization *i*'s extra-IO trade in year *t*,
$T_{i,t}$ denotes organization *i*'s total trade in year *t* (*i*'s total imports plus total exports),
$T_{w,t}$ denotes the world's total trade in year *t* (the world's total imports plus total exports).

The index for *Trade introversion* is:

- equal to minus one in the case of no intra-IO trade;
- equal to zero if the organization's weight in its own trade is equal to its weight in the trade of the rest of the world (geographic neutrality);

[5] Trade intensity has a notable outliner in the OECS. This is due to the extremely small size of the RO (2017 estimate of total population is 615,724, and of total GDP $6.7 billion) as compared to any other RO in the dataset. This leads to very large mean and SD measures for *trade intensity*.

- equal to one in the case of no extra-IO trade.

The value for *Trade introversion* ranges from -1 to +1.

Each index has its supporters and detractors (for a discussion of pros and cons, see Iapadre and Plummer 2011). The most popular index is intra-IO trade share, but it is vulnerable to the ups and downs of economic cycles, which expand or contract an IO's intra-trade value irrespective of whether there has been trade integration. The trade intensity index avoids this problem. However, it has limitations that complicate comparison across IOs: first, the maximum value is a decreasing function of an IO's total trade, which implies that a given value stands for different things for different-sized IOs; second, it is characterized by range asymmetry, in that the range below unity is much smaller than above, which can bias comparison of IOs with values on either side of unity. The trade introversion index "can be proposed as the most satisfactory measure of intra-regional trade intensity" (Iapadre and Plummer 2011, p. 108). However, it is the most complex and least intuitive of the three. Hence the three measures approach trade interdependence quite differently.

Power asymmetry

In constructing our measure of power asymmetry, we updated the Composite Index of National Material Capabilities (CINC) version 5.0 (Singer 1988; Singer et al. 1972). The traditional CINC measure summarizes military expenditure, military personnel, energy consumption, iron and steel production, urban population, and total population annually from 1950 to 2012. Our alternative measure summarizes total population, total GDP, and military expenditure annually from 1950 to 2019. Our measure is the ratio in material capabilities of the largest member state to the sum of all member states of the IO. *Sources for GDP*: Feenstra, Robert C., Robert Inklaar and Marcel P. Timmer (2015), "The Next Generation of the Penn World Table", *American Economic Review*, 105(10), 3150-3182, available for download at www.ggdc.net/pwt. *Sources for population*: World Bank. "Population, total." The World Bank Group. *Sources for military expenditures*: SIPRI. "SIPRI Military Expenditure Database 1949-2019."

Democracy

Democracy is the average democratic quality of an IO, calculated as the mean of the V-Dem democracy score for each member state of an IO in a given year. A country-year V-Dem score is itself the mean of five indices—the *electoral democracy index, liberal democracy index, participatory democracy index, deliberative democracy index, and egalitarian democracy index*—which are scaled from 0 to 1. The five components are drawn from the V-Dem dataset. *Source*: Coppedge, Michael et al. "V-Dem Dataset v10" Varieties of Democracy (V-Dem) Project.

GDP

We use two GDP measures: the mean Gross Domestic Product (GDP) per capita of the member states of an IO in a given year and a measure that gauges the standard deviation of the annual GDP per capita (i.e. dispersion). Observations are for the period 1950-2019 and are derived from the latest version of the Penn World Table (PWT 10.0, released June 18, 2021), and use the *output-side real GDP at current PPPs* (in mil. 2017US$). *Sources*: Feenstra, Robert C., Robert

Inklaar and Marcel P. Timmer (2015), "The Next Generation of the Penn World Table", *American Economic Review*, 105(10), 3150-3182, available for download at www.ggdc.net/pwt.

Preference heterogeneity
We estimate the incongruence among the members of an IO in voting in the UN assembly. Voting is arrayed on a single dimension that reflects state positions toward the US-led liberal order. Votes are aggregated by UN session. The unit is the absolute distance between country A and country B's posterior mean ideal-point estimates. The measure is the annual average by IO of the absolute distance between ideal points for all dyads of an IO's member states between 1950 to 2019. Annual measure. *Source*: the variable absidealdiff as calculated by Bailey, Strezhnev, and Voeten (2017).

Appendix D: Descriptive statistics and correlation matrix

Descriptive statistics

	Mean	SD	Min	Max
Delegation	0.189	0.154	0	0.652
Policy scope	9.255	5.840	0	25
Contract	0.655	0.475	0	1
Politicization	0.209	0.465	0	3.186
Democratic IO	0.485	0.500	0	1
Age	26.920	17.530	1	70
Members	2.283	0.746	1.099	4.007
Democracy	0.434	0.227	0.074	0.796
GDP per capita	16808	15024	583	85406
GDP dispersion	9703	11937	277	102785
Year	1992	17.6	1950	2019
Power asymmetry	4.151	3.552	1	22.720
Trade interdependence	24.720	22.060	0.800	75.510
Cold War	0.406	0.491	0	1
Preference heterogeneity	0.396	0.286	0.00147	1.841
Trade interdependence (trade intensity)	16.750	56.970	0.394	1233.500
Trade interdependence (trade introversion)	0.673	0.281	-0.444	0.999
Power asymmetry (CINC)	4.914	4.234	1	23.380
Number of observations = 1928				

Correlation matrix

	Delegation	Policy scope	Contract	Politicization	Democratic IO	Age	Members	Democracy	GDP dispersion	GDP per capita SD	Year	Power asymmetry	Trade interdependence	Cold War	Preference heterogeneity	Trade intensity	Trade introversion	Power asymmetry (CINC)
Delegation	1.000																	
Policy scope	0.374	1.000																
Contract	0.208	0.641	1.000															
Politicization	0.289	0.340	0.053	1.000														
Democratic IO	0.186	-0.269	-0.352	0.034	1.000													
Age	0.292	0.232	0.021	0.279	0.295	1.000												
Members	0.147	0.055	-0.089	0.237	-0.042	0.188	1.000											
Democracy	0.263	-0.184	-0.348	0.068	0.907	0.351	-0.087	1.000										
GDP dispersion	0.166	-0.152	-0.352	0.190	0.392	0.378	-0.094	0.410	1.000									
GDP per capita SD	0.004	-0.020	-0.160	0.198	-0.106	0.176	0.117	-0.191	0.636	1.000								
Year	0.267	0.386	0.037	0.380	-0.013	0.595	0.164	0.071	0.270	0.186	1.000							
Power asymmetry	0.004	0.069	-0.088	0.263	-0.007	0.125	0.684	-0.038	-0.082	0.042	0.146	1.000						
Trade interdependence	0.291	-0.264	-0.359	0.267	0.539	0.120	0.381	0.567	0.329	-0.039	-0.064	0.375	1.000					
Cold War	-0.226	-0.336	-0.021	-0.345	-0.033	-0.487	-0.121	-0.107	-0.202	-0.102	-0.841	-0.130	0.018	1.000				
Preference heterogeneity	-0.178	-0.231	-0.245	0.174	0.057	-0.135	0.317	-0.017	-0.119	0.042	-0.033	0.349	0.274	0.049	1.000			
Trade intensity	0.023	0.143	0.162	-0.067	-0.006	-0.039	-0.164	0.004	-0.174	-0.136	0.052	-0.152	-0.183	-0.024	-0.063	1.000		
Trade introversion	0.243	0.295	0.321	0.097	-0.022	0.016	-0.099	0.088	-0.230	-0.440	0.196	-0.073	0.136	-0.202	-0.145	0.273	1.000	
Power asymmetry (CINC)	-0.026	0.069	-0.001	0.193	-0.018	0.130	0.704	-0.072	-0.161	0.023	0.093	0.918	0.271	-0.082	0.377	-0.154	-0.102	1.000

Appendix E: An instrumental variables (IV) approach using prior history, contract, and policy scope

We need to address the potential endogeneity between contract and policy scope. To do so we carry out a IV approach that substitutes IO contract (the treatment) with an instrument that is strongly associated with the type of contract (relevance of the instrument) but exogenous to both contract and policy scope, and that is arguably unrelated to policy scope except through the treatment (exclusion restriction). One such exogenous factor is the presence or absence of a shared political past among the IO members, such as past common statehood or former coexistence in the same colonial empire. An IO is categorized as having *Historical ties* when at least two-thirds of the founding members 1) were once members of the same federation, or 2) share experience of membership within—and resistance to—a colonial empire.[6] It seems plausible that open-ended contracts are easier to agree if the parties involved can tap into a normative commonality that was forged before IO cooperation. So here is a two-step argument whereby a shared political past creates the normative conditions for states to settle on an open-ended contract, and this open-ended contract in turn provides the needed flexibility to adapt an IO's policy portfolio to evolving circumstances.

A parsimonious way to model this is through a two-stage model (Wooldridge 2002) that, first, evaluates the extent to which the instrument *Historical ties* explains the treatment *Contract*, and second, estimates the effect of the treatment *Contract* on changing *Policy scope*. We are mindful that for *Historical ties* to be considered a valid instrument, two assumptions should be reasonably evident. First, the relevance of the instrument implies that IOs with historical ties should be more likely to have open-ended contracts than those without (first stage). Second, the exclusion restriction implies that historical ties should show evidence of affecting *Policy scope* only through the type of contract. We use the stata command xtivreg2 (Schaffer 2010). As Model 1 in Table E1 shows, *Historical Ties* is statistically strong. Moreover, according to the Stock–Yogo test, we can reject the null hypothesis of weak instrumentation at the strictest threshold of 10%. In the first-stage model, *Historical ties* is the *only* variable that consistently explains *Contract* under a range of controls. In a bivariate fixed effects regression, *Historical ties* explains about one-sixth of the overall variation in *Contract* ($R^2=0.17$). We can therefore say with relative confidence that shared political history does indeed shape the type of IO contract states conclude, therefore fulfilling the assumption of the relevance of the instrument and demonstrating *Historical ties* is a strong IV. In the second stage (Model 2), instrumented *Contract* is significantly associated with *Policy scope* (p=0.059). Hence the analysis substantiates that a) *Historical ties* explains *Contract*, and b) *Contract* mediates change in an IO's *Policy scope*.

[6] We model the impact of history by multiplying *Historical ties* by the age of the IO, and by imposing a decay factor on the intuition that the longer ago these historical ties ceased to exist the dimmer the shared memories or institutions. The formula for calculating the decay factor is y= e^ (-(inception of IO-final year of historical ties)/25) if *Historical ties*=1; otherwise y=0. *Historical dynamic* is then y*age of IO.

Table E1. Two-stage fixed effects OLS regression

	First stage DV=Contract dynamic	Second stage DV=Policy scope
Historical ties	0.766***	
	(0.168)	
Contract dynamic $_{t-1}$ (instrumented)		0.101*
		(0.053)
Democracy $_{t-1}$	15.901	8.828
	(21.774)	(6.610)
Members $_{t-1}$	3.701	2.331
	(5.341)	(2.228)
Power asymmetry $_{t-1}$	-0.020	-0.483**
	(1.096)	(0.207)
GDP per capita $_{t-1}$	-2.526	-0.493
	(3.309)	(0.895)
GDP dispersion $_{t-1}$	2.491	0.294
	(1.710)	(0.445)
Trade interdependence $_{t-1}$	0.337	-0.051
	(0.238)	(0.077)
Preference heterogeneity $_{t-1}$	1.620	-1.216
	(4.995)	(1.174)
Year count	1.472***	-0.059
	(0.177)	(0.090)
R^2 (within)	0.944	0.546
Kleibergen-Paap Wald rk F-statistic[a]	20.86	
F-statistic		10.94
Wald F p-value	0.023	0.000
Stock-Yogo test for weak instrument[b]	H_0 rejected	

Note: N=1708 IO-year (41 IOs) for 1950-2015. Fixed effects estimation with standard errors clustered by IO. *** p<0.01, ** p<0.05, * p<0.1. *GDP per capita* and *GDP dispersion* are standardized for easier interpretation.

[a] The Kleibergen-Paap Wald F-statistic probes the null hypothesis that the excluded instruments are weakly associated with the endogenous variable (Kleibergen and Paap 2006). The higher the F-statistic, the more the instrument is well correlated with the endogenous variable.

[b] The Stock-Yogo test probes the null hypothesis that the instrument is weak, where weakness is estimated as the size of the bias of the IV estimator relative to the Kleibergen-Paap F test at a set threshold (Stock and Yogo 2005). Rejecting the null hypothesis at the strictest threshold of 10% signifies that the instrument is *not* weak, and here the Stock-Yogo F-statistic at the 10% level is well below the Kleibergen-Paap Wald F statistic (16.38< 20.86).

The second stage also provides the opportunity to seek evidence of the validity of the exclusion restriction. Our sample includes three IOs (Benelux, Caricom, and IGAD), that have historical ties, were founded with closed contracts, and changed to open-ended contracts during the IO's lifetime. If the exclusion restriction holds, then these IOs should behave similarly to other IOs with the same contract. That is to say, in the years that they existed under closed contracts, they should display patterns of policy scope similar to other IOs with closed contracts; in the years that they function under open-ended contracts, they should display patterns of policy scope similar to other IOs with open-ended contracts. As Fig. F1 below illustrates, this is clearly the case for all three IOs that change contract during their lifetime. Benelux changes from fixed to open-ended 12 years after its founding (1960), Caricom 5 years after founding (1973), and IGAD 10 years after founding (1996). Furthermore, in all three cases the policy scope of the IO is within one standard deviation from the mean for both IOs with closed contracts (before transition), and IOs with open-ended contracts (after transition). Therefore, we argue that within our instrumented variable model, *Historical ties* affects policy scope through the type of contract of the IO, which corroborates the assumption of the exclusion restriction.

Figure E1. Policy scope and age of IO, comparison of open-ended and closed contracts

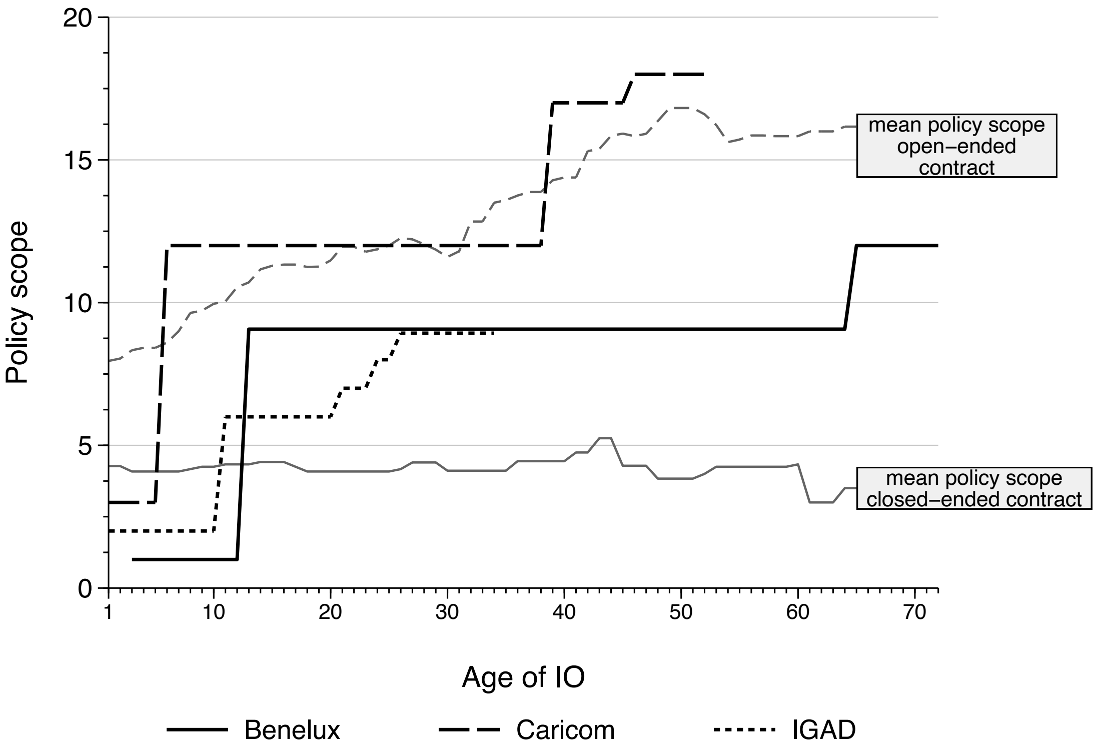

Note: Benelux moves from a closed- to an open-ended contract 12 years after founding, Caricom 5 years after founding and IGAD 10 years after founding.

Appendix F: Robustness checks

Table F1. Time-series cross-sectional analysis of *Policy Scope*

VARIABLES	(1)<br>Random effects	(2)<br>Lagged dependent variable
Policy scope $_{t-1}$		0.916***
		(0.014)
Open-ended contract $_{t-1}$	1.754*	-0.576**
	(0.924)	(0.250)
Age $_{t-1}$	0.002	-0.003
	(0.041)	(0.005)
Open-ended contract $_{t-1}$ * Age $_{t-1}$	0.137***	0.011***
	(0.032)	(0.004)
Members $_{t-1}$	1.728	0.081
	(1.896)	(0.219)
Democracy $_{t-1}$	5.950	0.685
	(5.732)	(0.760)
GDP per capita $_{t-1}$	-0.523	-0.046
	(0.833)	(0.085)
GDP dispersion $_{t-1}$	0.355	0.063
	(0.430)	(0.050)
Power asymmetry $_{t-1}$	-0.400**	-0.031
	(0.202)	(0.028)
Trade interdependence $_{t-1}$	-0.055	-0.008
	(0.065)	(0.010)
Cold War $_{t-1}$	-0.986**	-0.281***
	(0.474)	(0.084)
Preference Heterogeneity $_{t-1}$	-0.963	0.034
	(1.024)	(0.200)
Constant	3.621	1.152*
	(3.996)	(0.622)
Observations	1708	1708
R-squared		0.932
Number of IOs	41	41

Robust standard errors in parentheses; *GDP per capita* and *GDP dispersion* are standardized for easier interpretation.

*** $p<0.01$, ** $p<0.05$, * $p<0.1$

Table F2. Time-series cross-sectional analysis of *Delegation* (continuous measure of democracy)

VARIABLES	(1)<br>Random effects	(2)<br>Lagged dependent variable	(3)<br>Lagged dependent variable (reduced model)	(4)<br>Continuous democracy variable	(5)<br>Excluding PIF, SPC, GCC, and ECCAS
Delegation $_{t-1}$		0.881***	0.892***		
		(0.023)	(0.021)		
Policy scope $_{t-1}$	0.014***	0.002***	0.001***	0.017*	0.012***
	(0.004)	(0.001)	(0.001)	(0.010)	(0.004)
Politicization $_{t-1}$	-0.028	0.008		-0.133	-0.033
	(0.041)	(0.009)		(0.131)	(0.043)
Policy scope $_{t-1}$ * Politicization $_{t-1}$	0.004	-0.000		0.012	0.004
	(0.003)	(0.001)		(0.009)	(0.003)
Democratic IO $_{t-1}$	-0.016	-0.002			-0.048
	(0.046)	(0.007)			(0.045)
Democratic IO $_{t-1}$ * Policy scope $_{t-1}$	-0.003	-0.001			-0.002
	(0.004)	(0.001)			(0.004)
Democratic IO $_{t-1}$ * Politicization $_{t-1}$	0.096**	-0.000			0.105**
	(0.043)	(0.008)			(0.041)
Democratic IO $_{t-1}$ * Policy scope $_{t-1}$ * Politicization $_{t-1}$	-0.008**	-0.000			-0.008**
	(0.003)	(0.001)			(0.003)
Members $_{t-1}$	-0.026	-0.003	-0.004	-0.045	-0.030
	(0.027)	(0.005)	(0.005)	(0.033)	(0.033)
Power asymmetry $_{t-1}$	0.006	0.001	0.001	0.006	0.004
	(0.004)	(0.001)	(0.001)	(0.004)	(0.005)
GDP per capita $_{t-1}$	-0.030*	-0.005*	-0.005*	-0.039**	-0.059**
	(0.016)	(0.002)	(0.003)	(0.017)	(0.024)
GDP dispersion $_{t-1}$	0.007	0.002	0.003	0.010	0.019
	(0.009)	(0.002)	(0.002)	(0.009)	(0.016)
Year $_{t-1}$	0.002***	0.000	0.000	0.003***	0.003***
	(0.001)	(0.000)	(0.000)	(0.001)	(0.001)
Trade interdependence $_{t-1}$	0.001	-0.000	-0.000	0.000	-0.001
	(0.001)	(0.000)	(0.000)	(0.001)	(0.001)
Cold War $_{t-1}$	0.011	-0.005***	-0.005**	0.009	0.009
	(0.009)	(0.002)	(0.002)	(0.010)	(0.010)
Preference Heterogeneity$_{t-1}$	-0.039*	-0.003	-0.005	-0.050**	-0.039*
	(0.020)	(0.005)	(0.005)	(0.019)	(0.021)
Democracy (continuous) $_{t-1}$			0.001	-0.098	
			(0.019)	(0.157)	
Democracy (continuous) $_{t-1}$ * Policy scope $_{t-1}$				-0.007	
				(0.018)	
Democracy (continuous) $_{t-1}$* Politicization $_{t-1}$				0.299	
				(0.224)	
Democracy (continuous) $_{t-1}$* Policy scope $_{t-1}$* Politicization $_{t-1}$				-0.023	
				(0.014)	
Constant	-4.514***	-0.225	-0.163	-5.309***	-6.638***
	(1.472)	(0.232)	(0.246)	(1.729)	(1.754)
Observations	1,708	1,708	1,708	1,708	1,533
R-squared		0.915	0.914	0.561	0.584
Number of IOs	41	41	41	41	37

Robust standard errors in parentheses; *GDP per capita* and *GDP dispersion* are standardized for easier interpretation. *** p<0.01, ** p<0.05, * p<0.1

Figure F1. Effect of *Policy Scope* on *Delegation* at various levels of politicization for non-democratic IOs and democratic IOs (continuous measure of democracy)

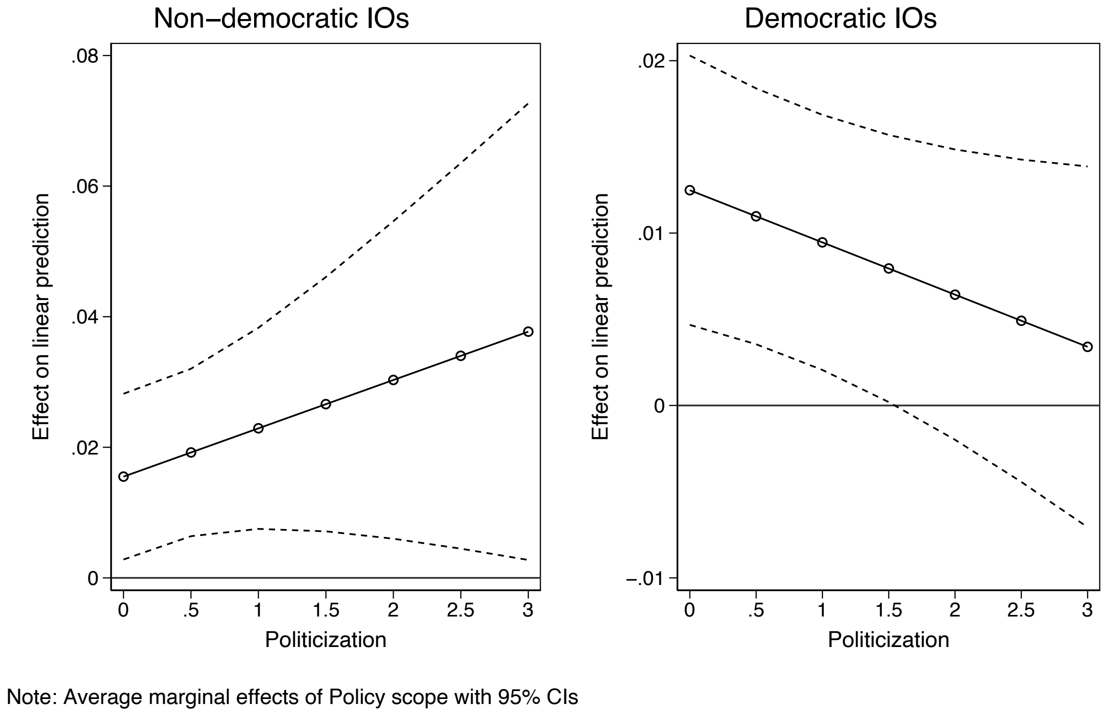

Note:
We constructed Figure F1 by first defining high and low values of the V-Dem democracy score as being one standard deviation above or below the mean. Thus, the left panel with the average marginal effects for non-democratic IOs corresponds to those IOs that are one standard deviation below the mean of the V-Dem score and the right panel corresponds to those IOs that are one standard deviation above the mean of this measure.

References

Bailey, M. A., Strezhnev, A., & Voeten, E. (2017). Estimating dynamic state preferences from United Nations voting data. *Journal of Conflict Resolution*, *61*(2), 430–456.

Balassa, B. (1961). *The theory of economic integration*. Homewood: Irwin.

Boehmer, C., Gartzke, E., & Nordstrom, T. (2004). Do intergovernmental organizations promote peace? *World Politics*, *57*(1), 1–38.

Haftel, Y. (2013). Commerce and institutions: Trade, scope, and the design of regional economic organizations. *Review of International Organizations*, *8*(3), 389–414.

Haftel, Y., & Hofmann, S. (2017). Institutional authority and security cooperation within regional economic organizations. *Journal of Peace Research*, *54*(4), 484–498.

Hooghe, L., Lenz, T., & Marks, G. (2019). *A theory of international organization*. Oxford: Oxford University Press.

Hooghe, L., Marks, G., Lenz, T., Bezuijen, J., Ceka, B., & Derderyan, S. (2017). *Measuring international authority: A postfunctionalist theory of governance*. Oxford: Oxford University Press.

Iapadre, L., & Plummer, M. (2011). Statistical measures of regional trade integration. In de Lombaerde, P., Flores, R. G. & Iapadre, L. (eds.), *The regional integration manual* (pp. 98–123). London: Routledge.

Kleibergen, F., & Paap, R. (2006). Generalized reduced rank tests using the singular value decomposition. *Journal of Econometrics*, *133*(1): 97–126.

Lundgren, M., Squatrito, T., & Tallberg, J. (2018). Stability and change in international policy-making: A punctuated equilibrium approach. *Review of International Organizations*, *13*(1), 547–572.

Marks, G., Lenz, T., Ceka, B., & Burgoon, B. (2014). Discovering Cooperation: A Contractual Approach to Institutional Change in Regional International Organizations. Robert Schumann Center for Advanced Studies (RSCAS) Working Paper Series, Nr. 2014/65, Florence: European University Institute.

Schaffer, Mark. 2020. xtivreg2: Stata module to perform extended IV/2SLS, GMM and AC/HAC, LIML and k-class regression for panel data models. *Statistical Software Components.*

Singer, J. D. (1988). Reconstructing the Correlates of War dataset on material capabilities of states, 1816-1985. *International Interactions*, *14*(2), 115–132.

Singer, J. D., Bremer, S., & Stuckey, J. (1972). Capability distribution, uncertainty, and major power war, 1820-1965. In Russett, B. (ed.), *Peace, war, and numbers* (pp. 19–48). Beverly Hills, CA: Sage.

Stock, J. & Yogo, M. (2005). Asymptotic distributions of instrumental variables statistics with many instruments. In Andrews, D. W. K. & Stock, J. (eds.), *Identification and inference for econometric models: Essays in honor of Thomas Rothenberg* (pp. 109–120). Cambridge: Cambridge University Press.

Tallberg, J., Sommerer, T., Squatrito, T., & Jönsson, C. (2014). Explaining the transnational design of international organizations. *International Organization*, *68*(4), 741–774.

Wooldridge, J. M. (2002). *Econometric analysis of cross section and panel data*. Cambridge, MA: MIT Press.
